# Demographics, health literacy and health locus of control beliefs of Australian women who take complementary medicine products during pregnancy and breastfeeding: A cross‐sectional, online, national survey

**DOI:** 10.1111/hex.13414

**Published:** 2021-12-23

**Authors:** Larisa A. J. Barnes, Margaret I. Rolfe, Lesley Barclay, Kirsten McCaffery, Parisa Aslani

**Affiliations:** ^1^ Faculty of Medicine and Health, School of Pharmacy and University Centre for Rural Health The University of Sydney Sydney New South Wales Australia; ^2^ Faculty of Medicine and Health, University Centre for Rural Health and School of Public Health The University of Sydney Lismore New South Wales Australia; ^3^ Faculty of Medicine and Health, School of Public Health The University of Sydney Sydney New South Wales Australia; ^4^ Faculty of Medicine and Health, School of Pharmacy The University of Sydney Sydney New South Wales Australia

**Keywords:** breast feeding, complementary medicine products, dietary supplements, health literacy, health locus of control, herbal medicine, pregnancy

## Abstract

**Background:**

Pregnant and breastfeeding women's use of complementary medicine products (CMPs) is common, and possibly associated with autonomous health care behaviours. However, the health literacy levels and health locus of control (HLOC) beliefs of women who use CMPs in pregnancy and lactation have not been previously assessed in a large Australian sample.

**Aim:**

The aim of this study is to determine the health literacy levels and HLOC beliefs of women who use CMPs in pregnancy and lactation and determine the types of CMPs used.

**Methods:**

A cross‐sectional, national, online survey of Australian pregnant or breastfeeding women aged 18 years and older, and currently using CMPs was conducted.

**Results:**

A total of 810 completed surveys (354 pregnant and 456 breastfeeding women) were analysed. Most had adequate functional health literacy levels (93.3%). Health care practitioners (HCPs) HLOC mean scores were the highest for the sample, followed by Internal HLOC beliefs mean scores. Almost all (*n* = 809) took at least one dietary supplement, the most popular being pregnancy and breastfeeding multivitamins, iron supplements and probiotics. Use was generally in line with clinical recommendations, except for low rates of iodine supplementation. Herbal medicine use was lower for the total sample (57.3%, *n* = 464), but significantly higher (*p* < .0001) for the breastfeeding cohort, with consumers taking one to four herbal medicines each. The most popular herbs were raspberry leaf, ginger, peppermint and chamomile (pregnant respondents) and chamomile, ginger and fenugreek (breastfeeding respondents).

**Conclusions:**

Respondents were health literate, with high scores for Internal and HCP HLOC scales, suggesting that they are likely to demonstrate self‐efficacy, positive health behaviours and work well in partnership with HCPs. HCPs can facilitate discussions with pregnant and breastfeeding women using CMPs, while considering women's health literacy levels, health beliefs and goals.

## 1 BACKGROUND

Many Australians' health care practices include the use of complementary medicine (CM), including complementary medicine products (CMPs) like dietary supplements and herbal medicines.^1^, ^2^, ^3^, ^4^, ^5^ During pregnancy and lactation, some CMPs like iodine and folic acid supplements have been shown in clinical trials to improve outcomes for the mother and baby, and so are routinely prescribed or recommended as part of mainstream evidence‐based biomedical maternity care.^6^ These recommendations are also endorsed by CM practitioners in Australia.^7^, ^8^, ^9^ As such, the definition of a CMP can vary according to context, and the prescriber or recommender. Self‐prescription of CMPs, including herbal medicines and dietary supplements like folic acid, is also common.^8^, ^10^ The following operational definition of CMPs was used in this study, and was based on documents from the Pharmacy Guild of Australia,^11^ the Australian Government Department of Health and Ageing,^12^ the Australian National Health Medical Research Council,^13^ The Royal Australian and New Zealand College of Obstetricians and Gynaecologists [RANZCOG]^6^ and several Australian research papers and textbooks,^7^, ^8^, ^9^, ^14^, ^15^ and aligns with international definitions of CMPs^16^, ^17^, ^18^, ^19^, ^20^  (File S4):

*CMPs are products like herbal medicines and vitamin and mineral supplements and probiotics. Some vitamins and minerals (e.g., iron, folate or iodine supplements) may be recommended by your doctor or other health care practitioner and have a scientific evidence base. Other CMPs like some herbal medicines may have traditional uses but may not have been scientifically researched*.

Women's use of CMPs in pregnancy and lactation has been associated with women's desire to positively enhance their own health and that of their babies,^9^, ^21^, ^22^ including the treatment of common conditions of pregnancy (e.g., nausea and vomiting of pregnancy, preparation for labour)^9^, ^10^, ^21^ and lactation (e.g., blocked ducts, mastitis and concerns with breastmilk supply).^9^, ^22^, ^23^ Mothers' CM use has also been associated with having tertiary levels of education^7^, ^10^, ^23^, ^24^, ^25^, ^26^, ^27^, ^28^, ^29^ and higher income or employment levels,^10^, ^23^, ^25^, ^28^, ^30^ and being nonsmokers.^26^, ^29^, ^30^ Previous Australian qualitative research found good functional health literacy levels to be linked to CMP use in pregnancy and lactation.^22^, ^31^, ^32^ Previous research has also noted that women's use of CM and CMPs helps facilitate their self‐determination, autonomy and control over health during pregnancy and lactation.^33^, ^34^, ^35^, ^36^, ^37^, ^38^

The Health Locus of Control Form C (MHLC‐C)^39^, ^40^ measures *Internal, Doctors (operationalized as health care practitioners [HCPs] in this study), Other People or Chance Locus of Control* beliefs,^40^, ^41^ with results indicating where a respondent believes control of her health, and in respect to pregnancy and lactation, where responsibility for the health of her unborn or breastfeeding children, lies.^41^ Studies focusing on Health Locus of Control (HLOC) beliefs^39^, ^42^ in pregnancy^43^, ^44^ and breastfeeding^45^ have found that higher Internal HLOC beliefs are associated with several different aspects of health and self‐efficacy, including positive self‐care behaviours in mothers with gestational diabetes^46^; choosing to birth in midwifery‐led, low‐intervention birthing units over obstetrician‐led medical wards^44^; breastfeeding self‐efficacy and success^45^; and positive mental health pre‐^47^ and postnatally.^45^ In general populations, higher Internal HLOC beliefs in healthy adults have been associated with increased use of CM therapies and CMPs,^48^, ^49^, ^50^, ^51^ and healthy behaviours including regular exercise.^48^ Previous research^9^, ^52^, ^53^, ^54^, ^55^, ^56^ has revealed that pregnant and breastfeeding women's use of CMPs is linked to beliefs that CMP use is health‐promoting for both themselves and their babies; however, the HLOC beliefs of mothers using CMPs have not been measured before. Measuring HLOC beliefs in women who use CMPs during pregnancy and lactation could help confirm the type/s of control beliefs associated with this use, and confirm whether self‐efficacy, or dependence on others, chance or HCPs influences women's CMP use during pregnancy. This would help and inform the practices of HCPs working in maternity care around the use of CMPs.

Maternal health literacy can be described as ‘the cognitive and social skills that determine the motivation and ability of women to gain access to, understand and use information in ways that promote and maintain their health and that of their children’.^57^ Good health literacy encourages healthy pregnancy and postpartum behaviours, and is a vital component of understanding and using the information to make health‐promoting decisions, including decisions about medicines used.^9^, ^58^ Despite the potential impact that poor health literacy could have on many aspects of health and health care choices during pregnancy and lactation,^58^ the effects of maternal health literacy on women's reproductive health and CMP use is under‐researched.^9^ Previous research has confirmed the high prevalence of CMP use in pregnancy and lactation^7^, ^8^, ^10^, ^23^, ^24^, ^26^, ^59^ and raised concerns regarding maternal health literacy and the ability to make safe decisions regarding CMP use in pregnancy and lactation.^1^, ^60^, ^61^, ^62^ Nevertheless, this previous research has not included measurements of health literacy in pregnant and breastfeeding respondents with respect to the use of CMPs.

As part of a larger, national cross‐sectional study investigating factors influencing women's decision‐making regarding the use of CMPs in pregnancy and lactation, this paper reports on the women's health literacy levels, HLOC beliefs and the types of CMPs used and compares the use of CMPs by the pregnant and breastfeeding cohorts.

## 2 METHODS

### 2.1 Ethical approval

Ethical approval for the study was obtained from The University of Sydney Human Research Ethics Committee, approval number 2018/1010. The survey questionnaire was completed and submitted online, and completion of the questionnaire was taken as consent to participate. The survey questionnaires were completed anonymously, and no identifying data such as name or date of birth were collected. The Participant Information Statement (PIS) informed participants of these considerations. Additionally, the PIS clearly stated that participants could withdraw their consent to participate in the study at any time before submitting their completed surveys, but that because all the data were collected anonymously, it would not be possible to extract submitted data once completed surveys had been submitted. The PIS also outlined an incentive to participate: At the end of the survey, respondents were given the option of entering their email addresses to go into the draw to win an iPad mini® and/or to receive a summary of the overall results of the study. If they chose either of these options, they were automatically redirected to a separate survey so that their email addresses were not linked to the information gathered in the study survey.

### 2.2 Survey design

A national, cross‐sectional, online, anonymous, self‐administered questionnaire was designed and set up using the Qualtrics^63^ platform. The questionnaire (File S1) comprised of 70 questions and took approximately 20 min to complete. The completed Checklist for Reporting Results of Internet E‐Surveys (CHERRIES)^64^, ^65^ appears in File S2.

### 2.3 Inclusion criteria

The inclusion criteria for the study were aged 18 years or over, currently pregnant and/or breastfeeding, currently taking one or more CMPs and living in Australia. Three eligibility screening questions were used at the beginning of the survey.

### 2.4 Patient or public contribution

This study was designed by a multidisciplinary team of HCPs and researchers without direct public involvement. However, the survey items were informed by data from earlier qualitative research with the same population.^22^, ^31^, ^32^ The pilot questionnaire was designed by the research team, all of whom have experience of pregnancy and motherhood, and three of whom have the clinical experience of working with pregnant or breastfeeding women as a naturopath (L. A. J. B.), pharmacist (P. A.) and midwife (L. B.), respectively. The questionnaire was piloted by several lay‐women volunteers who fulfilled the study inclusion criteria. Volunteers piloted the questionnaire on tablets, mobile telephones and laptops. Each volunteer trialled the questionnaire twice (once as a pregnant participant and once as a breastfeeding participant). They were invited to comment on its usability and relevance, and their data were not included in the final data analysis. Volunteers were asked to comment on the ease and usability of the questionnaire, as well as their understanding of the questions, which helped confirm face validity.^66^ Feedback was generally positive, with volunteers reporting that the survey made sense and was easy to understand, flowed well and covered topics they expected in a survey on CMP use in pregnancy and breastfeeding (content validity). The participants did not suggest any wording changes to the questions, nor did they suggest any additional questions. Furthermore, their understanding of the questions and purpose of the study was aligned with our understanding. It took between 17 and 25 min for each of the volunteers to complete the questionnaire. The first 20 completed questionnaires were also examined to ascertain how long it took for respondents to complete the survey. The Qualtrics data showed that the minimum length of time taken was 14 min and the maximum time was 30 min (average time was 22 min).

### 2.5 Sample size calculations

Sample sizes were based on calculated populations of pregnant and breastfeeding women. The number of registered births in Australia in one year was used as a proxy number for the population of Australian pregnant women (*n* = 311,104).^67^ Data reporting the number of Australian infants receiving any breastmilk were used as a proxy number for the number of breastfeeding mothers in Australia (*n* = 163,478).^68^ Prevalence sample size calculations with finite population corrections were performed using the online tool http://sampsize.sourceforge.net/iface/ for all outcome variables. The populations for pregnancy and breastfeeding were calculated separately. All calculations used a precision of 5% and a confidence interval of 95%. The calculated sample sizes were 384 pregnant respondents and 384 breastfeeding respondents (768 complete surveys).^69^

### 2.6 Recruitment

Recruitment occurred entirely online and primarily through posts generated from a Facebook page specific to the research project, and have been described elsewhere.^70^ Paid promoted posts (‘boosted’ posts) were used to advertise the study to potential participants. Purposive and snowball recruitment occurred by requesting posts to be shared on relevant Australian Facebook pages and by sharing nonboosted posts through the research team's own social network connections. All posts contained a link to the survey in the Qualtrics platform. No Internet Protocol (IP) addresses or other identifying data were retained as part of the survey data. The survey recruitment period lasted 10 weeks (6 July–17 September 2019).

### 2.7 Measures

The complete survey is presented in File S1. The following sections outline the specific sections relevant to this paper.

#### 2.7.1 Demographic characteristics

Demographic questions included age, smoking status, pregnancy or breastfeeding status, number of children, gestational age of the child (pregnant participants), age of the breastfeeding child (breastfeeding participants), marital status, postcode of residence (to assess rurality), weekly household income, education levels, country of birth of the respondent and countries of birth of her parents and  the main language spoken at home.

#### 2.7.2 CMP use

The operational definition of CMPs (see Section Background, 1) was provided to respondents at several points in the survey. Respondents were asked to indicate the dietary supplements and/or the herbal medicines that they currently consumed from lists of CMPs commonly reported as being used in pregnancy or lactation.^22^, ^31^, ^32^ An ‘other’ category with the option of including free‐text responses was included.

#### 2.7.3 Health literacy

Health literacy levels were measured using two validated health literacy tools: the single‐item health literacy screening question,^71^ which measures respondents' risk of inadequate health literacy using a single, simple question, and the Newest Vital Sign (UK version),^72^ which measures functional health literacy levels in under 3 min using six questions about a nutrition label. Use of the two validated health literacy tests helped confirm the consistency of the results by facilitating comparisons between respondents' risk of inadequate health literacy and their functional health literacy skills.

#### 2.7.4 Health locus of control beliefs

The validated 18‐item MHLC‐C^39^, ^40^ was used to test whether high *Internal, Doctors (operationalized as ‘Health Care Practitioners’), Other People, or Chance Locus of Control* health beliefs influenced respondents' CMP use decision‐making in pregnancy and lactation. The MHLC‐C was developed to be adapted for use with people living with any disease‐ or health‐related condition.^39^, ^41^ For the purposes of this study, ‘health and well‐being during pregnancy’ and ‘health and well‐being as a breastfeeding mother’ were substituted for the word ‘condition’ in the MHLC‐C for the pregnant and breastfeeding participants, respectively.

### 2.8 Data analysis

Data were analysed using IBM SPSS Statistics V24 and Excel. Data were screened and incomplete surveys were removed as per the protocol, which outlined that incomplete surveys would be removed before analysis, pending the receipt of at least 768 complete surveys to enable meaningful data analysis^73^ (see sample size calculations). Surveys marked ‘complete’ in Qualtrics, indicating that the respondent had progressed through all 70 survey items, were included in analyses, provided that at least 75% of the items were completed. Descriptive analyses, followed by *χ* ^2^ tests, were carried out for all demographic, health literacy and CMP use data to examine differences between the pregnant and breastfeeding respondents. Missing data were not included in the statistical analyses. Statistical significance was defined as a *p* < .05.

The research hypotheses tested were that there would be no statistically significant differences between the two cohorts (pregnant and breastfeeding women) in the total number of dietary supplements or herbal medicines taken; that both cohorts would be similar in their functional health literacy levels and that there would be no differences between the two groups in the numbers of women at risk of inadequate health literacy; and that both cohorts would have similar HLOC scores for all four subscales. Poisson regression analysis was performed to model the count data for dietary supplements and herbal medicines, respectively, to observe whether there were significant differences in the numbers taken between the pregnant and breastfeeding respondents.

#### 2.8.1 Health literacy levels

For the single‐item health literacy screening question *How confident are you filling out medical forms by yourself?*,^71^ respondents answering ‘somewhat’ or ‘a little bit’ or ‘not at all’ were considered to be at risk of inadequate health literacy. Those answering ‘extremely’ or ‘quite a bit’ confident were not considered to be at risk of inadequate health literacy.^71^ For the *Newest Vital Sign*, respondents who scored 0–1 correct (out of six questions) were considered to have a high likelihood of limited functional health literacy skills.^72^, ^74^ Those who scored 2–3 were considered to be at risk of inadequate functional health literacy skills, and those who scored 4–6 correct were considered to have adequate functional health literacy skills.^72^, ^74^

#### 2.8.2 Health locus of control beliefs

Means for each subscale of the MHLC‐C were calculated for the two cohorts, hence providing scores on the original 1–6 subscales. To examine differences between the results for the breastfeeding and pregnancy cohorts, and calculate estimated marginal means of measure, a repeated‐measures analysis of variance analysis was performed for the four HLOC subscales.

## 3 RESULTS

### 3.1 Responses collected

A total of 1418 women were enroled in the survey. Of these, 168 respondents were excluded as they did not fulfil the eligibility criteria, and a further 440 incomplete surveys were removed. A total of 810 completed surveys (57.1%) were collated for analysis.

### 3.2 Demographics

Of the 810 responding women, 325 (40.1%) were currently pregnant; 456 (56.3%) were currently breastfeeding; and 29 (3.6%) were currently both pregnant and breastfeeding. For all data analyses, the respondents who were currently both pregnant and breastfeeding were included in the pregnant respondents' group, resulting in two sub‐samples (cohorts) being ‘currently pregnant’ (*n* = 354, 43.7% of the total sample) or ‘currently breastfeeding’ (*n* = 456, 56.3%). Almost half of the respondents (*n* = 363, 44.8%) reported having two or more children, and 369 (45.6%) were pregnant with (*n* = 161, 22%) or breastfeeding their first child (*n* = 208, 28.4%; Table 1).

**Table 1:** Pregnancy‐ and breastfeeding‐related characteristics of the sample

	Number of respondents (*n*)	Relative frequency (%)
Pregnant or breastfeeding status (*n* = 810)		
Currently pregnant	325	40.1
Currently breastfeeding	456	56.3
Currently pregnant and breastfeeding	29	3.6
Total	810	100
If currently pregnant (includes those currently pregnant and breastfeeding) (*n* = 354)		
First trimester (0–12 weeks)	58	16.4
Second trimester (13–27 weeks)	140	39.5
Third trimester (28–42 weeks)	156	44.1
Total	354	100
If currently breastfeeding, age of breastfed child (*n* = 456)		
0–2 months	104	22.8
3–5 months	106	23.2
6–8 months	67	14.7
9–11 months	43	9.4
12–15 months	47	10.3
16–18 months	19	4.2
19–23 months	30	6.6
Over 2 years old	40	8.8
Total	456	100
Number of children (*n* = 810)		
Pregnant with her first child	161	22.0
Breastfeeding her first child	208	28.4
2 Children	270	36.9
3 Or more children	93	12.7
Total	732	100
Missing data	78	

Respondents ranged in age from 19 to 53 years, with the mean age being 33.8 years (SD = 4.6) and the median age being 34.0 years. Other demographic data are summarized in Table 2. There were no significant differences between the demographic characteristics of the two cohorts, except for income levels, where pregnant respondents had significantly higher income (Pearson's *χ* ^2^ = 16.430, *p* = .021; Table 2).

**Table 2:** Demographic characteristics of the sample

	Pregnant respondents (*n *= 354)	Relative frequency (%)	Breastfeeding respondents (*n* = 456)	Relative frequency (%)	Whole sample (*n* = 810)	Relative frequency (%)	Pearson's *χ* ^2^ value	*p* Value
Smoking status								
Currently smokes	4	1.2	5	1.3	9	1.2	35.043	0.371
Does not currently smoke	322	98.2	390	98.2	712	98.2		
Prefer not to respond	2	0.6	2	0.5	4	0.6		
Total	328	100	397	100	725	100		
Missing data	26		59		85			
Marital status								
Single	5	1.5	6	1.5	11	1.5	1.603	0.659
Married or in de facto relationship	322	97.3	391	97.8	713	97.5		
Separated or divorced	1	0.3	2	0.5	3	0.4		
Other	3	0.9	1	0.3	4	0.5		
Total	331	100	400	100	731	100		
Missing data	23		56		79			
Highest education level								
Year 10 (school certificate)	4	1.2	5	1.3	9	1.2	2.718	0.91
Year 12 (high school certificate) or equivalent	9	2.7	10	2.5	19	2.6		
Certificate 1–4	13	3.9	23	5.8	36	4.9		
Diploma	24	7.2	31	7.8	55	7.5		
Associate diploma	5	1.5	8	2	13	1.8		
Bachelor's degree	126	38	156	39	282	38.5		
Postgraduate studies at university	149	44.9	163	40.8	312	42.6		
Other	2	0.6	4	1	6	0.8		
Total	332	100	400	100	732	100		
Missing data	22		56		78			
Approximate weekly household income level ($AUD)								
No income	2	0.6	0	0	2	0.3	16.43	0.021^a^
$1–$456 per week	6	1.8	10	2.5	16	2.2		
$457–$960 per week	27	8.2	40	10	67	9.2		
$961–$1616 per week	64	19.3	115	28.8	179	24.5		
$1617–$2489 per week	101	30.5	106	26.6	207	28.4		
$2490–$5036 per week	74	22.4	62	15.5	136	18.6		
$5037 or above per week	13	3.9	11	2.8	24	3.3		
I prefer not to answer	44	13.3	55	13.8	99	13.6		
Total	331	100	399	100	730	100		
Missing data	23		57		80			
Main language spoken at home								
English	324	97.9	385	96.5	709	97.3	3.141	0.208
Other	7	2.1	14	3.5	20	2.7		
Total	331	100	399	100	729	100		
Missing data	23		57		81			
Country of birth								
Australia	258	77.7	324	81.2	582	79.6	17.203	0.07
The United Kingdom	18	5.4	14	3.5	32	4.4		
New Zealand	10	3	5	1.3	15	2.1		
China	3	0.9	1	0.3	4	0.5		
India	3	0.9	2	0.5	5	0.7		
The Philippines	6	1.8	0	0	6	0.8		
Vietnam	0	0	3	0.8	3	0.4		
South Africa	5	1.5	8	2	13	1.8		
Malaysia	3	0.9	3	0.8	6	0.8		
Germany	1	0.3	2	0.5	3	0.4		
Other	25	7.5	37	9.3	62	8.5		
Total	332	100	399	100	731	100		
Missing data	22		57		79			
Participation by Australian state or territory								
Australian Capital Territory	19	5.7	16	4	35	4.8	15.371	0.052
New South Wales	133	40.2	185	46.6	318	43.7		
Northern Territory	3	0.9	3	0.8	6	0.8		
Queensland	59	17.8	58	14.6	117	16.1		
South Australia	24	7.3	21	5.3	45	6.2		
Tasmania	14	4.2	17	4.3	31	4.3		
Victoria	53	16	72	18.1	125	17.2		
Western Australia	26	7.9	25	6.3	51	7		
Total	331	100	397	100	728	100		
Missing	23		59		82			
Urban or rural residence according to the ASGS_RA_2016 categories^75^ ^,^ b								
Major cities of Australia	235	71	266	66.8	501	68.7	10.711	0.057
Inner regional Australia	71	21.5	94	23.6	165	22.6		
Outer regional Australia	22	6.6	32	8	54	7.4		
Remote and very remote Australia	3	0.9	6	1.5	9	1.2		
Total	331	100	398	100	729	100		
Missing	23		58		81			

^a^ Pregnant respondents had significantly higher incomes than breastfeeding respondents.

^b^ ASGS_RA_2016 categories = Australian Statistical Geography Standard^75^—Remoteness area categories as used by the Australian Bureau of Statistics.

### 3.3 CMP use

All respondents reported taking at least one CMP. The two main types of CMPs used by the sample were dietary supplements and herbal medicines. Almost the whole sample reported taking dietary supplements (*n* = 808). Herbal medicine use was much lower: 464 respondents (57.3%) reported that they took herbal medicines (*n* = 186, 52.5% of pregnant respondents and *n* = 278; 61.0% of breastfeeding respondents).

#### 3.3.1 Dietary supplements

Only two breastfeeding respondents (0.4%) reported that they did not take any dietary supplements. All pregnant respondents reported taking at least one dietary supplement (Table 3). The most popular dietary supplements taken by the sample overall were pregnancy and breastfeeding multivitamins (*n* = 566, 70.0%), followed by probiotics (*n* = 306, 37.9%) and iron supplements (*n* = 278, 34.4%). Iodine supplementation in the sample was low, with only 41 (11.6%) pregnant respondents and 33 (7.3%) breastfeeding respondents reporting taking iodine (Table 3).

**Table 3:** Types of dietary supplements used by the respondents

Dietary supplement^a^	Pregnant (*n* = 354)	Relative frequency (%)	Breastfeeding (*n* = 456)	Relative frequency (%)^b^	Total sample (*n* = 810)	Relative frequency (%)^b^
Pregnancy or breastfeeding multivitamin	282	79.7	284	62.6	566	70.0
Iron	140	39.5	138	30.4	278	34.4
Probiotics (e.g., acidophilus)	111	31.4	195	43.0	306	37.9
Vitamin D	109	30.8	128	28.2	237	29.3
Folic acid	84	23.7	35	7.7	119	14.7
Omega 3 supplements	75	21.2	116	25.6	191	23.6
Vitamin C	56	15.8	41	9.0	153	18.9
Other	47	13.3	77	17.0	124	15.3
Calcium	44	12.4	53	11.7	97	12.0
Iiodine	41	11.6	33	7.3	74	9.2
Zinc	32	9.0	56	12.3	88	10.9
Vitamin B_12_	32	9.0	36	7.9	68	8.4
B vitamins	27	7.6	55	12.1	82	10.1
Vitamin B_6_	20	5.6	23	5.1	43	5.3
Evening primrose oil	6	1.7	6	1.3	12	1.5

^a^ Respondents could choose more than one option.

^b^ *n* = 2 missing data.

Respondents reported taking between 1 and 18 supplements each, with most (*n* = 450, 62.8%) taking between one and three supplements (File S3, Table AF3.1). Pregnant respondents took an average of 3.4 supplements each (median = 3.0), and breastfeeding respondents took an average of 3.5 supplements each (median = 3.0). Poisson regression analysis showed that there were no significant differences between the numbers of dietary supplements taken by pregnant respondents versus breastfeeding respondents (Wald *χ* ^2^ = 3695.806, *p* = .413).

#### 3.3.2 Herbal medicines

Only 186 (52.5%) pregnant respondents and 278 (61.0%) breastfeeding respondents reported taking herbal medicines. For the whole cohort, the herbal medicines used most frequently were ginger (*n* = 120, 14.8%), chamomile (*n* = 118, 14.6%), peppermint (*n* = 104, 12.8%) and raspberry leaf (*n* = 100, 12.3%; Table 4). The most frequently reported herbs taken by pregnant respondents were raspberry leaf (*n* = 58, 16.4%), followed by ginger (*n* = 57, 16.1%), peppermint (*n* = 51, 14.4%) and chamomile (*n* = 40, 11.3%). Chamomile was the most frequently reported herb taken by the breastfeeding cohort (*n* = 78, 17.1%), followed by ginger (*n* = 63, 13.8%) and fenugreek (*n* = 61, 13.4%; Table 4).

**Table 4:** Types of herbal medicines used by the respondents

Herbal medicine^a^	Pregnant respondents (*n* = 354)	Relative frequency (%)^a^	Breastfeeding respondents (*n* = 456)	Relative frequency (%)^b^	Total sample (*n* = 810)	Relative frequency (%)^b^
Raspberry leaf	58	16.4	42	9.2	100	12.3
Cranberry	15	4.2	14	3.1	29	3.6
Echinacea	25	7.1	55	12.1	80	9.9
Ginger	57	16.1	63	13.8	120	14.8
Peppermint	51	14.4	53	11.6	104	12.8
Chamomile	40	11.3	78	17.1	118	14.6
Fenugreek	4	1.1	61	13.4	65	8.0
St. Mary's thistle (milk thistle)	2	0.6	19	4.2	21	2.6
Dong quai	2	0.6	4	0.9	6	0.7
Shatavari	0	0.0	10	2.2	10	1.2
Herbal extracts or teas from my health practitioner	11	3.1	31	6.8	42	5.2
Other (please specify)	24	6.8	57	12.5	81	10.0
I do not currently take any herbal medicines	168	47.5	178	39.0	346	42.7

^a^ Results are not mutually exclusive—respondents could choose all herbal medicines that applied.

^b^ All respondents answered this question.

Respondents who reported using herbs were taking between 1 and 15 herbal medicines each, with most taking between one and four (File S3, Table AF3.2). Pregnant respondents took an average of 2.1 herbal medicines each (median = 2.0), and breastfeeding respondents took an average of 2.7 herbal medicines each (median = 2.0).

Poisson regression analysis showed that the number of herbal medicines taken reported by the breastfeeding respondents was significantly higher than the number taken by the pregnant respondents (Wald *χ* ^2^ = 589.584, *p* < .0001).

### 3.4 Health literacy

Results from the single‐item health literacy question^71^ indicated that only 28 respondents (3.8%) were at risk of inadequate health literacy (File S3, Table AF3.3). There were no significant differences between the numbers of pregnant and breastfeeding respondents at risk of inadequate health literacy (Pearson's *χ* ^2^ = 6.814, *p* = .146; File S3, Table AF3.3).

Results from the *Newest Vital Sign* ^72^, ^74^ indicated that 682 respondents (93.3%) had adequate functional health literacy skills File S3, Table AF3.4). There were no significant differences between the numbers of pregnant and breastfeeding respondents with adequate functional health literacy (Pearson *χ* ^2^ = 2.523, *p* = .283; File S3, Table AF3.4).

### 3.5 Health locus of control

For the whole sample, *HCPs HLOC* had the highest mean scores (above 4.0 for both cohorts), followed by *Internal HLOC*, scoring above 3.5 for both cohorts (Table 5). Both *Chance* and *Other People HLOC* means scored considerably lower (Table 5). This indicated that the whole sample felt that HCPs had substantial control over their pregnancy or breastfeeding health, although this was significantly higher for the pregnant cohort. The high Internal HLOC scores also indicated that the whole sample had strong beliefs in their own abilities to control their health.

**Table 5:** Health locus of control (HLOC) means for the four subscales

		*N* respondents	Missing (*n*/810)	Mean	Std. deviation	Std. error mean	*p* Value
Health care practitioners HLOC	Pregnant	332	78	4.68	0.78	0.04	<.0001*
	Breastfeeding	400		4.26	0.97	0.05	
Internal HLOC	Pregnant	332	79	3.66	0.80	0.04	<.0001***
	Breastfeeding	399		4.01	0.84	0.04	
Chance HLOC	Pregnant	332	79	2.89	0.88	0.05	<.0001**
	Breastfeeding	399		2.55	0.9	0.05	
Other people HLOC	Pregnant	331	80	3.26	0.82	0.05	.933
	Breastfeeding	399		3.25	0.88	0.04	

* Pregnant respondents had a significantly higher health care practitioner HLOC mean score.

** Pregnant respondents had a significantly higher Chance HLOC mean score.

*** Breastfeeding participants had a significantly higher Internal HLOC mean score.

Significant differences between the two cohorts were found for Internal HLOC, HCP HLOC and Chance HLOC mean scores, but not for Other People HLOC mean scores, Wilks' *λ* = 0.33, *F* (3,726) = 483.09, *p* < .0001 (Table 5 and File S3, Figure AF3.1). The high mean Internal HLOC scores indicated that the whole sample had strong beliefs in their own abilities to control their health. Although the mean scores for the Chance HLOC were much lower than for the Internal HLOC and HCPs HLOC, significantly more pregnant respondents felt that chance or fate influenced their health compared to the breastfeeding respondents. The Other People HLOC results show that the whole sample did not feel that Other People had significant control over their pregnancy or breastfeeding health.

## 4 DISCUSSION

The objectives of this paper were to investigate the health literacy levels and HLOC beliefs of Australian women who use CMPs in pregnancy and lactation. Differences in the types of CMPs used by the two cohorts in the sample were investigated, as were differences in health literacy levels and HOLC beliefs. The respondents were highly educated, health literate and engaged in health‐promoting behaviours. They were unique compared to previous samples of Australian pregnant and breastfeeding women in general, and those using CMPs in pregnancy and lactation, in three main areas: parity (number of children), breastfeeding history and health literacy levels. While primiparity is associated with CMP use in some Australian^8^, ^26^, ^28^, ^30^, ^76^ studies, nearly half of the respondents (44.8%) reported having two or more children. To our knowledge, and following careful literature scrutiny, this is the first Australian study that has shown this result. The high proportion of women breastfeeding an infant over 5 months of age in the breastfeeding cohort (*n* = 246, 53.9%) is also notable because the rates of breastfeeding in Australia fall far below^77^, ^78^ the optimal recommendations of exclusive breastfeeding for 6 months, with the gradual introduction of family foods and continued breastfeeding for 12–24 months and beyond.^79^, ^80^, ^81^ This is important, as Australian breastfeeding rates are known to dramatically drop from 6 months of age, when approximately 60% of Australian babies receive any breastmilk, to only 7.4% of Australian babies receiving any breastmilk between 19 and 24 months of age.^68^ The vast majority of respondents were health literate, and a large proportion (*n* = 594, 81.1%) were university educated. Similar results regarding parity, long‐term breastfeeding and high health literacy were found in the qualitative research that informed the survey.^32^

The respondents were somewhat similar in language background and age to the general population of Australian pregnant and breastfeeding women.^67^, ^82^ However, apart from English being the main language spoken at home, their cultural and ethnic diversity did not reflect that of the wider Australian population. Similarly, the sample's country of birth was not reflective of the wider Australian population as 79.6% reported being born in Australia, which is around 13% higher than the 2016 census data.^82^ That said, a few interesting parallels with previous work can be seen. Higher levels of education,^7^, ^10^, ^23^, ^24^, ^25^, ^26^, ^27^, ^28^, ^29^ and income or employment,^10^, ^23^, ^25^, ^28^, ^30^ and being nonsmokers^26^, ^29^, ^30^ have previously been significantly associated with CM use in pregnancy and lactation. One pregnancy study found that living in urban areas was significantly associated with herbal medicine use in pregnancy.^10^ However, in the wider Australian community, mixed results have been shown, with some studies noting higher use of CM in rural areas,^3^, ^83^, ^84^, ^85^ and others showing higher use in urban areas.^86^ There were not enough participants from outer regional, remote or very remote Australia areas in this study to examine differences in rural and urban participation and CMP use, but the results did show that CMP use in pregnancy and lactation occurs throughout all Australian regions.

## 5 CMP USE

### 5.1 Use of dietary supplements

Women's use of dietary supplementation was generally in line with recommendations that aim to ensure optimal health of the mother and baby pre‐ and postnatally,^6^ especially regarding multivitamin, folic acid, iron and Vitamin D supplementation. The high use of pregnancy and breastfeeding multivitamins (70.0% of the total sample) is in line with current recommendations of RANZCOG, who state that ‘[m]ost proprietary pregnancy and lactation multivitamin preparations are adequate for the majority of pregnancies [and will cover dietary needs for vitamin D and folic acid]. The commonest exceptions will be the vegetarian/vegan needing additional iron and women for whom a high dose (5 mg) of folic acid or pharmacological doses of vitamin D are recommended’.^6^ ^(p8)^ Previous research has found high rates of dietary supplementation in Australian women to be prevalent, especially use of multivitamin^7^, ^8^, ^87^ and iron supplements.^7^, ^8^ Notably, 30.8% of the pregnant cohort and 28.2% of the breastfeeding cohort took vitamin D (29.3% of the total sample); similar rates were found in Shand et al.'s^7^ survey of pregnant women in Sydney. Vitamin D supplementation in pregnancy is recommended if pathology testing shows deficiencies, to prevent low vitamin D levels in the neonate and decrease the mother's risk of developing osteoporosis.^6^ It may also be recommended postnatally as vitamin D deficiency has been associated with postpartum depression in some mothers.^88^, ^89^

Iodine supplementation by the sample was low, with only 9.2% of the total sample reporting taking iodine. These low rates are of concern, and are much lower than the 23% adherence noted in Malek et al.'s^87^ South Australian study. Iodine is necessary for the physical and mental health of the pre‐ and postnatal mother,^88^ and to prevent some forms of delayed cognitive function in infants.^13^, ^87^ Mild iodine deficiency in Australia is common due to low iodine levels in our soils, low uptake of fortified foods and reduced use of iodized salt,^13^, ^87^ and has resulted in the mandatory fortification of breads with iodized salt since 2009.^90^ However, this fortification is unlikely to meet the increased needs for iodine in pregnancy and lactation, and the National Health and Medical Research Council (Australia) recommends that all pregnant and lactating women supplement with 150 μg of iodine daily.^6^ It would seem that public health messages regarding iodine supplementation pre‐ and postnatally are having limited success with the survey sample, especially considering the high health literacy levels of the respondents.

### 5.2 Use of herbal medicines

Respondents reported using far fewer herbal medicines in comparison to dietary supplements. Previous research has found that only 40%–50% of Australian pregnant women use herbal medicines during pregnancy,^25^ and other research has found much lower rates.^7^ The herbs used are similar to those reported previously in pregnancy^21^, ^26^, ^55^ and lactation.^23^, ^91^ While there are numerous calls for more research into the safety of herbal medicine use in pregnancy and lactation,^92^, ^93^ the most popular herbs used by pregnant respondents (raspberry leaf, ginger, peppermint and chamomile) do have long histories of traditional use around the world.^14^, ^94^, ^95^ Ginger has also been the subject of numerous clinical trials, and is considered safe at appropriate doses to help with nausea and vomiting of pregnancy.^96^, ^97^ Raspberry leaf is not considered to be dangerous to the foetus after the second trimester, although evidence for its traditional use to facilitate normal labour at the end of the third trimester is limited.^14^, ^98^ Peppermint and chamomile teas are commonly used to help alleviate nausea and soothe indigestion both pre‐ and postnatally,^14^, ^94^, ^95^ although some concerns exist with the ingestion of peppermint oil or concentrated peppermint extracts during pregnancy.^14^ Fenugreek is commonly used around the world as a galactagogue,^91^, ^92^, ^95^, ^99^ and recent meta‐analysis has confirmed its ability to increase breastmilk supply in lactating women.^100^ Ginger and chamomile are considered safe to use in breastfeeding and are traditionally used to aid digestion and relaxation.^23^, ^101^ Only one case study exists of a woman who experienced increased milk supply after ingesting chamomile.^101^

### 5.3 Health literacy

Regarding health literacy, several social determinants of health are associated with good health literacy, including higher education, income and general literacy levels,^72^, ^102^, ^103^ so it is not surprising that the survey sample had adequate health literacy levels. Combined with access to Internet resources and computer literacy, some of these determinants of health are also known to positively influence potential respondents' willingness to participate in online surveys,^104^, ^105^ as is good health literacy.^106^ As the sample cannot be seen to be a representative sample, it is difficult to infer whether the respondents' health literacy levels are likely to be present across all Australian women who use CMPs in pregnancy or lactation, or are just characteristic of those motivated to participate in this study. However, previous research has noted that women's use of CM, including CMPs, is to facilitate self‐determination, autonomy and control over their health during pregnancy and lactation.^33^, ^34^, ^35^, ^36^, ^37^, ^38^ Autonomy and a feeling of control over one's own health are known to have positive health benefits for individuals as they are more likely to be engaged in their health care, health care decisions and positive health behaviours, with flow‐on effects for physical, emotional, cultural and social health.^103^, ^107^, ^108^, ^109^ The majority of women in this study were not at risk of poor health literacy, and had adequate functional health literacy skills. These positive results in the health literacy tests may also be indicative of good maternal health literacy^57^ levels in the sample. Good maternal health literacy may also help explain the sample's positive health behaviours during pregnancy and the postpartum period, including the majority being nonsmokers,^110^ their use of prenatal folic acid supplements,^58^, ^111^ higher than average breastfeeding rates^58^ and breastfeeding self‐efficacy.^112^

### 5.4 HLOC beliefs

The HLOC results yielded new insights into the health beliefs of Australian pregnant and breastfeeding mothers using CMPs. These results are valuable for HCPs working in maternity care to consider, especially considering that the multidimensional HLOC scales and theory are also often used to predict health behaviours.^41^, ^43^, ^44^ The entire survey sample demonstrated high HCP HLOC beliefs, followed by high Internal HLOC beliefs. This pairing of high beliefs has been noted in other studies involving pregnant and breastfeeding women.^44^ The high HCP HLOC indicated that respondents believed that HCPs had significant roles to play in determining their pregnancy or breastfeeding health. Additionally, their high Internal HLOC beliefs indicated that women believed that their pregnancy or breastfeeding health was largely dependent on their own behaviours. This pairing of high scores for both Internal and HCP HLOC scales suggests that respondents are likely to work well and in partnership with HCPs,^42^, ^44^ strongly adhere to recommendations from their HCPs^113^, ^114^ and demonstrate self‐efficacy in carrying out positive health behaviours.^41^, ^48^, ^114^ This, along with their adequate health literacy levels discussed above, could explain some of the sample's positive health behaviours, including taking CMPs (e.g., folic acid and iron supplements), as recommended by biomedical organisations like RANZCOG,^6^ and being predominantly nonsmokers.^110^, ^115^

Internal HLOC beliefs may be considered to be somewhat stable over a lifetime,^44^, ^116^ but HCPs can have significant impacts on the health outcomes of women in pregnancy, birth and the postnatal period.^43^, ^44^, ^81^, ^117^ In the medical arena, pregnancy and birth are commonly viewed within a ‘risk’ model, and the samples' high HCP HLOC beliefs may reflect a reliance on their HCPs to reduce the perceived risks associated with pregnancy and birth, and reflect a recognition that HCPs play an essential role in their health pre‐ and postnatally.^43^, ^44^ High Internal HLOC beliefs are considered predictive of positive mother–baby attachment pre‐ and postnatally, as well as positive, autonomous, self‐care behaviours.^46^, ^118^, ^119^, ^120^ This attachment is considered fundamental to both a woman's psychological adjustment to motherhood and the psychological health and development of the baby after birth and throughout early childhood.^118^, ^119^, ^120^ The high Internal HLOC beliefs of the women who participated in this study may also be indicative of their positive attachment to their unborn and breastfeeding children.

Interestingly, the breastfeeding cohort had significantly higher Internal HLOC mean scores compared to the pregnant cohort. This may reflect changes in health‐seeking behaviours from the prenatal to the postpartum period. The postpartum year is a time of great physical and psychosocial change for women, often associated with decreased self‐care as the focus shifts from care of self to care of the infant.^121^, ^122^ Optimal health in the postpartum year necessitates the positive use of learned life skills to promote self‐efficacy.^121^ The higher use of CMPs in the breastfeeding cohort, including higher use of herbal medicines, may be associated with breastfeeding mothers' attempts to promote their own health, and that of their babies. The higher Internal HLOC mean scores may also be associated with logistical issues of not having regular appointments with HCPs, as with routine pregnancy care. Another possible interpretation of the breastfeeding cohort's higher Internal HLOC scores involves considerations of breastfeeding self‐efficacy. Lawal and Idemudia^45^ examined breastfeeding self‐efficacy, HLOC beliefs and psychological well‐being of breastfeeding mothers and found that a strong Internal HLOC directly influenced a mother's sense of autonomy and breastfeeding self‐efficacy, psychological health and positive relations with others. A large proportion of the breastfeeding cohort in the survey study was breastfeeding babies 6 months and older, demonstrating a certain amount of breastfeeding success and self‐efficacy.

## 6 LIMITATIONS

Biases inherent in cross‐sectional, self‐administered, anonymous survey research are associated with online recruitment, including nonrepresentative sampling,^64^ self‐selection bias^64^, ^105^ and exclusion of women without access to the Internet,^73^, ^104^ or with low levels of proficiency in the English language. The results thus cannot be said to be representative of the entire population of Australian pregnant and/or breastfeeding mothers.^66^ However, the use of purposeful sampling was intentional. The survey did not aim to be representative, or to assess population prevalence of CMP use in pregnancy and lactation.^39^, ^41^, ^42^ The focus of recruitment was to ensure adequate numbers of respondents from the pregnant and breastfeeding cohorts across a broad range of regions to enable meaningful data analysis.

The homogeneity across the sample regarding education, income, English‐language proficiency and health literacy levels is another limitation as the full demographic variability of pregnant and breastfeeding women in the wider Australian community may not be represented in the sample. This homogeneity is an unusual finding, especially considering that previous research has shown only approximately 40% of the Australian population has adequate health literacy levels.^123^, ^124^, ^125^ However, the health literacy profile of *all* Australian women who use CMPs in pregnancy and breastfeeding remains unknown. There may be Australian women who use CMPs in pregnancy and lactation who did not participate, including women from more diverse language and cultural backgrounds. For the latter groups, CMP use may well be a cultural imperative.^52^, ^126^, ^127^, ^128^ Further research on CMP use in pregnant and breastfeeding women from different cultural backgrounds could be desirable. It would also be useful to see if women with lower health literacy levels use CMPs in pregnancy and lactation, and if their HLOC beliefs differ from the respondents' beliefs in this study.

Finally, it was not possible to present results from the entire 70‐question survey in this paper. Future publications will focus on the respondents' reasons for CMPs use, their information and recommendation sources and perceptions and beliefs around safety and CMP use in pregnancy and lactation.

## 7 CONCLUSIONS

A large proportion of the sample had adequate functional health literacy levels, and were not at risk of limited functional health literacy. Respondents' HLOC beliefs indicated that they were more likely to be engaged in positive health behaviours and would work well in partnership with their HCPs. Respondents' high use of dietary supplements was generally in line with formal biomedical recommendations, except for iodine supplementation. The trust in HCPs shown by the sample indicates that they would probably consider taking iodine supplements if their HCPs recommend taking them and explain why iodine is important to women's and babies' health. HCPs should consider these survey findings when interacting with pregnant and breastfeeding women who use CMPs. Maintaining a woman‐centred focus to help identify women's individual health values and goals and considering health literacy levels and HLOC beliefs will support communication and collaboration between the health professional and the client. The sample's health literacy and high Internal HLOC results also highlight the need to support women in attaining self‐efficacy, especially in the postpartum year and during breastfeeding.

## CONFLICT OF INTERESTS

The authors declare that there are no conflicts of interest.

## AUTHOR CONTRIBUTIONS

Larisa A. J. Barnes, Lesley Barclay, Kirsten McCaffery, Margaret I. Rolfe and Parisa Aslani all were involved in the conceptualisation and design of the study. Larisa A. J. Barnes, Margaret I. Rolfe and Parisa Aslani performed the formal statistical analysis. Larisa A. J. Barnes was responsible for administering the Facebook page set up for participant recruitment, and for monitoring the survey in the online Qualtrics platform. Parisa Aslani was responsible for securing research funding from the University of Sydney School of Pharmacy. Larisa A. J. Barnes drafted the entire paper, with support from Parisa Aslani and Lesley Barclay. Larisa A. J. Barnes, Parisa Aslani, Lesley Barclay and Margaret I. Rolfe critically reviewed the paper. All authors read and approved the final manuscript.

## Supporting information

Supporting information.

Supporting information.

Supporting information.

Supporting information.

## Data Availability

The data sets generated and analysed during the current study are not publicly available as participants did not consent to their survey data being shared. Additional details relating to other aspects of the data are available on reasonable request from the authors
