# Supplementary material for: Demographics, health literacy and health locus of control beliefs of Australian women who take complementary medicine products during pregnancy and breastfeeding: A cross‐sectional, online, national survey
Source: Health Expect. 2021 Dec 23;25(2):667–83. doi: 10.1111/hex.13414 (PMC8957740; doi:10.1111/hex.13414)
Supplement: Supplementary file 1 — Supporting information. [file HEX-25--s002.pdf]

Additional File 1. Complete survey (Excluding Participant Informatioon Statement)

Complementary medicine product use during pregnancy and breastfeeding

Survey Flow

Standard: intro (1 Question)
Block: Default Question Block (2 Questions)
Standard: Block 1 (62 Questions)

Branch: New Branch
If
If Are you currently Neither pregnant or breastfeeding Is Selected

EndSurvey: Advanced

Branch: New Branch
If
If 3. Do you currently take any complementary medicine products? CMPs are defined as the following:P... No Is Selected

EndSurvey: Advanced

Branch: New Branch
If
If Do you live in Australia? No Is Selected

EndSurvey: Advanced

Block: Default Question Block (10 Questions)
Standard: Block 4 (4 Questions)
Block: Default Question Block (10 Questions)
Block: Default Question Block (10 Questions)
Standard: Block 7 (0 Questions)
Standard: Default Block (1 Question)

Q1 Thank you for telling us about your use of complementary medicine products in pregnancy and breastfeeding.

Please complete the survey below.
You can return to complete the survey at any time. The progress bar at the top will tell you how much of the survey you have finished at any time.

Q3 Are you currently

- ○ Pregnant (1)
- ○ Breastfeeding (2)
- ○ Both pregnant and breastfeeding (3)
- ○ Neither pregnant or breastfeeding (4)

Skip To: End of Block If Q3 = 4
Skip To: Q66 If Q3 = 1
Skip To: Q66 If Q3 = 3
Skip To: Q23 If Q3 = 2

Display This Question:

If Q3 = 1

Or Q3 = 3

Q66 If you are currently pregnant, how many weeks pregnant are you?

- ◯ 0-12 weeks (1st trimester) (1)
- ◯ 13-27 weeks (2nd trimester) (2)
- ◯ 28-42 weeks (3rd trimester) (3)

Display This Question:

If Q3 = 2

Q23 If you are currently breastfeeding, how old is the child you are breastfeeding?

- ◯ 0-2 months (1)
- ◯ 3-5 months (2)
- ◯ 6-8 months (3)
- ◯ 9-11 months (4)
- ◯ 12-15 months (5)
- ◯ 16-18 months (6)
- ◯ 19-23 months (7)
- ◯ over 2 years old (8)

Q4 3. Do you currently take any complementary medicine products?

CMPs are defined as the following: Products like herbal medicines (e.g. in teas, tablets, capsules or extracts), or vitamin and mineral supplements (e.g. multivitamins, iodine supplements, fish oils or probiotics). Some vitamins and minerals have a scientific evidence base (e.g. iron, folic acid or iodine supplements), and may be recommended by your doctor

or other healthcare practitioner.   Some CMPs like some herbal medicines may have traditional uses but may not have been scientifically researched.

- ◯ Yes (1)
- ◯ No (2)

Skip To: End of Block If Q4 = 2

Q5 Do you live in Australia?

- ◯ Yes (1)
- ◯ No (2)

Skip To: End of Block If Q5 = 2

Skip To: Q6 If Q5 = 1

---

*

Q6 What is your postcode?

____

Q7 How many children do you have?

- ◯ I am pregnant with my first child (1)
- ◯ I am breastfeeding my first child (2)
- ◯ I have 2 children (3)
- ◯ I have 3 or more children (4)

Page Break

Q24 **We would like to know about the health care practitioners women trust during pregnancy and breastfeeding.**

Display This Question:

If Q3 = 1

Or Q3 = 3

Q8 **You and your chosen health care practitioners.**
As a **pregnant mother**, which one of the following health care practitioners do you **most trust** when seeking health care for **yourself** during pregnancy?

- ○ Naturopath or herbalist (1)
- ○ Midwife (2)
- ○ Integrative medical doctor (3)
- ○ Traditional Chinese (Oriental) Medicine practitioner (4)
- ○ Child and Family Health Nurse (9)
- ○ General Practitioner (GP) (5)
- ○ Obstetrician (6)
- ○ Pharmacist (7)
- ○ Other (please specify) (8) ____

Display This Question:

If Q3 = 2

Q9 **You and your chosen health care practitioners.**
As a **breastfeeding mother**, which one of the following health care practitioners do you **most trust** when seeking health care for **yourself** whilst breastfeeding?

- ○ Naturopath or herbalist (1)
- ○ Midwife (2)
- ○ Integrative medical doctor (3)
- ○ Traditional Chinese (Oriental) Medicine practitioner (4)
- ○ Child and Family Health Nurse (9)
- ○ General practitioner (GP) (5)
- ○ Obstetrician (6)
- ○ Pharmacist (7)
- ○ Other (please specify) (8) ____

Q10
About your dietary supplements

We would like to understand the supplements women use in pregnancy or when breastfeeding and why.

Examples of supplements include vitamin or mineral supplements, fish oils, and probiotics. Please tell us about the supplements you currently use.

Q11 I currently take the following types of supplements (please choose all that apply)

- ▢ pregnancy or breastfeeding multivitamin (1)
- ▢ B vitamins (2)
- ▢ Vitamin B6 (3)
- ▢ Vitamin B12 (4)
- ▢ Vitamin C (5)
- ▢ Iodine (6)
- ▢ Calcium (7)
- ▢ Vitamin D (8)
- ▢ Iron (9)
- ▢ Zinc (10)
- ▢ Folic Acid (11)
- ▢ Omega 3 supplements (e.g. fish oil capsules or flax seed oil) (12)
- ▢ Evening primrose oil (13)
- ▢ Probiotics (e.g. acidophilus) (14)
- ▢ Other (please tell us what other supplements you take) (15) ____
- ▢ I do not currently take any supplements (16)

Skip To: Q19 If Q11 = 16

Q13 I take my supplements for the following reasons (please choose all that apply)

- ☐ To help my baby grow healthily (1)
- ☐ To help get all the nutrition I need to be healthy (2)
- ☐ To help me stay healthy (3)
- ☐ To stimulate breastmilk production (7)
- ☐ My health care practitioner prescribed them to me (4)
- ☐ To treat a specific health condition (5)
- ☐ Other reason (6) ____

Q14 Who recommended that you take supplements? (please choose all who apply)

- ☐ Naturopath or Herbalist (1)
- ☐ Midwife (2)
- ☐ Integrative Medical Doctor (3)
- ☐ Traditional Chinese (Oriental) Medicine practitioner (4)
- ☐ Child and Family Health Nurse (14)
- ☐ General Practitioner (GP) (5)
- ☐ Obstetrician (6)
- ☐ Paediatrician (15)
- ☐ Pharmacist (7)
- ☐ Other health care practitioner (please specify what type) (8) ____
- ☐ Pharmacy or health food shop staff (9)
- ☐ A family member (10)
- ☐ Another pregnant or breastfeeding woman (11)
- ☐ No one - I self-prescribed (12)
- ☐ Another source recommended I take supplements (please specify what source) (13) ____

Q15 Please indicate which health care professionals you get information on your supplements from (please choose all who apply)

- ☐ Naturopath or Herbalist (1)
- ☐ Midwife (2)
- ☐ Integrative Medical Doctor (3)
- ☐ Traditional Chinese (Oriental) Medicine practitioner (4)
- ☐ Child and Family Health Nurse (10)
- ☐ General Practitioner (GP) (5)
- ☐ Obstetrician (6)
- ☐ Paediatrician (11)
- ☐ Pharmacist (7)
- ☐ Other health care practitioner (please specify what type) (8)

____

Q16 Please indicate where you get information on your supplements from people in your personal social circles and / or from media (please choose all that apply)

- ☐ Family members (1)
- ☐ Other pregnant or breastfeeding women (2)
- ☐ Internet (e.g. Google) (3)
- ☐ Pregnancy or parenting app (4)
- ☐ Newspapers or magazines (7)
- ☐ My own personal experience (5)
- ☐ Other (please tell us) (6) ____

Q17 Please indicate whether you get information on your supplements from the following (please choose all that apply)

- ☐ Published research studies (1)
- ☐ Information from hospital pamphlets (2)
- ☐ Government or hospital websites (3)
- ☐ Medication websites (e.g. MotherSafe) (4)
- ☐ Medication helplines (e.g. MotherSafe, NPS Medicines line) (5)
- ☐ Other (please tell us where) (6) ____

Q19

About your herbal medicines

We would like to understand the herbal medicines women use in pregnancy, or when breastfeeding, and why. Examples of herbal medicines include herbal teas, herbal extracts, tablets and capsules.

Please tell us about the herbal medicines you currently use

Q20 I currently take the following types of herbal medicines. (Please choose all that apply)

- ▢ Raspberry leaf (1)
- ▢ Cranberry (2)
- ▢ Echinacea (3)
- ▢ Ginger (4)
- ▢ Peppermint (5)
- ▢ Chamomile (6)
- ▢ Fenugreek (7)
- ▢ Fennel (8)
- ▢ St Mary's thistle (Milk thistle) (9)
- ▢ Dong quai (10)
- ▢ Shatavari (11)
- ▢ Herbal extracts or teas from my health practitioner (12)
- ▢ Other (please specify) (13) ____
- ▢ I do not currently take any herbal medicines (14)

Skip To: Q74 If Q20 = 14

Q68 Who recommended that you take herbal medicines? (please choose all who apply)

- ▢ Naturopath or herbalist (1)
- ▢ Midwife (2)
- ▢ Integrative Medical Doctor (3)
- ▢ Child and Family Health Nurse (14)
- ▢ Traditional Chinese (Oriental) Medicine practitioner (4)
- ▢ General Practitioner (GP) (5)
- ▢ Obstetrician (6)
- ▢ Paediatrician (15)
- ▢ Pharmacist (7)
- ▢ Other health care practitioner (please specify what type) (8) ____
- ▢ Pharmacy or health food shop staff (9)
- ▢ A family member (10)
- ▢ Another pregnant or breastfeeding woman (11)
- ▢ No one - I self-prescribed (12)
- ▢ Another source recommended I take herbal medicines (please specify what source) (13) ____

Q69 Please indicate where you get information on your herbal medicines (please choose all who apply)

- [ ] Naturopath or herbalist (1)
- [ ] Midwife (2)
- [ ] Integrative Medical Doctor (3)
- [ ] Child and Family Health Nurse (9)
- [ ] Traditional Chinese (Oriental) Medicine practitioner (4)
- [ ] General Practitioner (GP) (5)
- [ ] Obstetrician (6)
- [ ] Paediatrician (10)
- [ ] Pharmacist (7)
- [ ] Other health care practitioner (please specify what type) (8) ________________

Q70 Please indicate where you get information on your herbal medicines from people in your personal social circles and / or from media (please choose all that apply)

- ☐ Family members (1)
- ☐ Other pregnant or breastfeeding women (2)
- ☐ Internet (e.g. Google) (3)
- ☐ Pregnancy or parenting app (4)
- ☐ Newspapers or magazines (7)
- ☐ My own personal experience (5)
- ☐ Other (please tell us) (6) ____

Q71 Please indicate whether you get information on your herbal medicines from the following (please choose all that apply)

- ☐ Published research studies (1)
- ☐ Information from hospital pamphlets (2)
- ☐ Government or hospital websites (3)
- ☐ Medication websites (e.g. MotherSafe (4)
- ☐ Medication helplines (e.g. MotherSafe, NPS) (5)
- ☐ Other (please tell us where) (6) ____

Q72 What other information source did you use to get information on your herbal medicines?

○ No other information source (1)

○ I get information on my herbal medicines from (please specify) (2)

____

Q67 I take my herbal medicines for the following reasons (please choose all that apply)

- ▢ To help my baby grow healthily (1)
- ▢ To help get all the nutrition I need to be healthy (2)
- ▢ To help me stay healthy (3)
- ▢ My health are practitioner prescribed them to me (4)
- ▢ To treat a specific health condition (5)
- ▢ Other reason (6) ____

Q74 Other complementary medicines you may take

Display This Question:

If Q3 = 1

Or Q3 = 3

Q21 **Other complementary medicine products you take as a pregnant mother**

Display This Question:

If Q3 = 1

Or Q3 = 3

Q22 I currently take other complementary medicine products for specific pregnancy conditions such as (please choose all that apply)

- ☐ I take these complementary medicine products to treat morning sickness (please list) (1) ____

- ☐ I take these complementary medicine products to prepare for labour (please list) (2) ____

- ☐ I take these complementary medicine products for other pregnancy-related conditions (please list) (3) ____

- ☐ I do not currently take any other complementary medicine products for specific pregnancy conditions (4)

Display This Question:

If Q3 = 2

Q25 **Other complementary medicine products you take as a breastfeeding mother**

Display This Question:

If Q3 = 2

Q26 I currently take other complementary medicine products for specific breastfeeding conditions such as (please choose all that apply)

- ☐ I take these complementary medicine products to increase my breastmilk supply (please list) (1) ____
- ☐ I take these complementary medicine products to treat or prevent mastitis (please list) (2) ____
- ☐ I take these complementary medicine products for other breastfeeding-related conditions (please list) (3) ____
- ☐ I do not currently take any other complementary medicine products for specific breastfeeding conditions (4)

Q27 Do you take any other complementary medicine products?

- ○ Yes, please list them (1) ____
- ○ No (2)

Q28 **We would like to understand what information sources women trust the most with regards to complementary medicine products.**

If you wanted information on a complementary medicine product, which of the following information sources would you trust most?

From the choices below, please choose your top 5 sources of trusted information for complementary medicine products. Please drag your choices into the box on the right.

My #1 most trusted source of information	My #2 most trusted source of information	My #3 most trusted source of information	My #4 most trusted source of information	My #5 most trusted source of information
______ Naturopath or herbalist (1)	______ Naturopath or herbalist (1)	______ Naturopath or herbalist (1)	______ Naturopath or herbalist (1)	______ Naturopath or herbalist (1)
______ Midwife (2)	______ Midwife (2)	______ Midwife (2)	______ Midwife (2)	______ Midwife (2)
______ Traditional Chinese (Oriental) Medicine practitioner (3)	______ Traditional Chinese (Oriental) Medicine practitioner (3)	______ Traditional Chinese (Oriental) Medicine practitioner (3)	______ Traditional Chinese (Oriental) Medicine practitioner (3)	______ Traditional Chinese (Oriental) Medicine practitioner (3)
______ Integrative medical practitioner (4)	______ Integrative medical practitioner (4)	______ Integrative medical practitioner (4)	______ Integrative medical practitioner (4)	______ Integrative medical practitioner (4)
______ General practitioner (GP) (5)	______ General practitioner (GP) (5)	______ General practitioner (GP) (5)	______ General practitioner (GP) (5)	______ General practitioner (GP) (5)
______ Child and Family Health Nurse (21)	______ Child and Family Health Nurse (21)	______ Child and Family Health Nurse (21)	______ Child and Family Health Nurse (21)	______ Child and Family Health Nurse (21)
______ Obstetrician (6)	______ Obstetrician (6)	______ Obstetrician (6)	______ Obstetrician (6)	______ Obstetrician (6)
______ Pharmacist (7)	______ Pharmacist (7)	______ Pharmacist (7)	______ Pharmacist (7)	______ Pharmacist (7)

_______ Pharmacy or health food shop personnel (8)	_______ Pharmacy or health food shop personnel (8)	_______ Pharmacy or health food shop personnel (8)	_______ Pharmacy or health food shop personnel (8)	_______ Pharmacy or health food shop personnel (8)
______ Other pregnant or breastfeeding mothers (9)	______ Other pregnant or breastfeeding mothers (9)	______ Other pregnant or breastfeeding mothers (9)	______ Other pregnant or breastfeeding mothers (9)	______ Other pregnant or breastfeeding mothers (9)
______ Family members or friends (10)	______ Family members or friends (10)	______ Family members or friends (10)	______ Family members or friends (10)	______ Family members or friends (10)
_______ Newspapers or magazines (20)	_______ Newspapers or magazines (20)	_______ Newspapers or magazines (20)	_______ Newspapers or magazines (20)	_______ Newspapers or magazines (20)
______ The Internet (11)	______ The Internet (11)	______ The Internet (11)	______ The Internet (11)	______ The Internet (11)
_______ Published research studies (12)	_______ Published research studies (12)	_______ Published research studies (12)	_______ Published research studies (12)	_______ Published research studies (12)
_______ Information from hospital pamphlets or websites (13)	_______ Information from hospital pamphlets or websites (13)	_______ Information from hospital pamphlets or websites (13)	_______ Information from hospital pamphlets or websites (13)	_______ Information from hospital pamphlets or websites (13)
_______ Medication websites or information helplines (e.g. MotherSafe) (14)	_______ Medication websites or information helplines (e.g. MotherSafe) (14)	_______ Medication websites or information helplines (e.g. MotherSafe) (14)	_______ Medication websites or information helplines (e.g. MotherSafe) (14)	_______ Medication websites or information helplines (e.g. MotherSafe) (14)
______ Other (please describe) (15)	______ Other (please describe) (15)	______ Other (please describe) (15)	______ Other (please describe) (15)	______ Other (please describe) (15)
______ Other (please describe) (16)	______ Other (please describe) (16)	______ Other (please describe) (16)	______ Other (please describe) (16)	______ Other (please describe) (16)
______ Other (please describe) (17)	______ Other (please describe) (17)	______ Other (please describe) (17)	______ Other (please describe) (17)	______ Other (please describe) (17)
______ Other (please describe) (18)	______ Other (please describe) (18)	______ Other (please describe) (18)	______ Other (please describe) (18)	______ Other (please describe) (18)

_______ Other (please describe) (19)	_______ Other (please describe) (19)	_______ Other (please describe) (19)	_______ Other (please describe) (19)	_______ Other (please describe) (19)

Display This Question:

If Q3 = 1

Or Q3 = 3

Q29 **Safety and complementary medicine use in pregnancy.**

The following questions are about your perceptions of safety regarding using complementary medicine products when you are pregnant. There are no right or wrong answers.
Complementary medicine products (CMPs) are - Products like herbal medicines, vitamin and mineral supplements and probiotics. - Some vitamins and minerals (e.g. iron, folic acid or iodine supplements) may be recommended by your doctor of other healthcare practitioner and have a scientific evidence base. - Some CMPs like some herbal medicines may have traditional uses but may not have been scientifically researched

Display This Question:

If Q3 = 1

Or Q3 = 3

Q33 ***When thinking about the complementary medicine products you take as a pregnant mother, please indicate how important you feel safety is for each of the following statements.***

	Not at all important (1)	Slightly important (2)	Moderately important (3)	Very important (4)	Extremely important (5)
It is important to me, that the complementary medicines I take are safe for my unborn child (1)	○	○	○	○	○
It is important to me, that the complementary medicines I take are safe for me (2)	○	○	○	○	○

Display This Question:

If Q3 = 1

Or Q3 = 3

Q54 ***When thinking about the complementary medicine products you take in pregnancy, please indicate how much you agree or disagree with each of the following statements.***

	Strongly disagree (1)	Disagree (2)	Somewhat disagree (3)	Neither agree nor disagree (4)	Somewhat agree (5)	Agree (6)	Strongly agree (7)
I have concerns about the safety of the complementary medicines I take for my unborn child (1)	o	o	o	o	o	o	o
I believe complementary medicines are more natural to use in pregnancy than medicines from the pharmacist or doctor (2)	o	o	o	o	o	o	o
I have concerns about the safety of the complementary medicines I take for myself (3)	o	o	o	o	o	o	o
I only take complementary medicine products that have been shown to be safe in pregnancy for the mother. (4)	o	o	o	o	o	o	o

I only take complementary medicine products that have been shown to help mothers have healthy pregnancies (5)	○	○	○	○	○	○	○
I only take complementary medicine products that have been shown to be safe for the unborn baby (6)	○	○	○	○	○	○	○
I believe complementary medicines are safer to use in pregnancy than medicines from the pharmacist or doctor (7)	○	○	○	○	○	○	○

Display This Question:

If Q3 = 2

Q31 **Safety and complementary medicine use in breastfeeding.**

The following questions are about your perceptions of safety regarding using complementary medicine products when you are breastfeeding. There are no right or wrong answers.

Complementary medicine products (CMPs) are
- products like herbal medicines, vitamin and mineral supplements and probiotics. - some vitamins and minerals (e.g. iron, folate or iodine supplements) may be recommended by your doctor of other healthcare practitioners and have a scientific evidence base. - Other CMPs like some herbal medicines may have traditional uses but may not have been scientifically researched

---

Display This Question:

If Q3 = 2

Q34 ***When thinking about the complementary medicine products you take as a breastfeeding mother, please indicate how important you feel safety is for each of the following statements.***

	Not at all important (1)	Slightly important (2)	Moderately important (3)	Very important (4)	Extremely important (5)
It is important to me, that the complementary medicines I take are safe for my breastfed child (1)	○	○	○	○	○
It is important to me, that the complementary medicines I take are safe for me (2)	○	○	○	○	○

Display This Question:

If Q3 = 2

Q32 ***Please think about your current use of complementary medicine products, and indicate how much you agree or disagree with the following statements.***

	Strongly agree (1)	Agree (2)	Somewhat agree (3)	Neither agree nor disagree (4)	Somewhat disagree (5)	Disagree (6)	Strongly disagree (7)
I have concerns about the safety of the complementary medicines I take for my breastfed child (8)	o	o	o	o	o	o	o
I believe that complementary medicines are more natural to use in breastfeeding than medicines from the pharmacist or doctor (2)	o	o	o	o	o	o	o
I have concerns about the safety of the complementary medicines I take for myself (9)	o	o	o	o	o	o	o
I only take complementary medicine products that have been shown to be safe for the breastfeeding mother (1)	o	o	o	o	o	o	o
I only take complementary medicine products that have been shown to help women breastfeed (3)	o	o	o	o	o	o	o

only take complementary medicine products that have been shown to be safe in breastfeeding for the breastfed baby (6)	○	○	○	○	○	○	○
I believe complementary medicines are safer to use in breastfeeding than medicines from the pharmacist or the doctor (5)	○	○	○	○	○	○	○
I only take complementary medicine products that have been shown to be safe for the breastmilk supply in breastfeeding. (7)	○	○	○	○	○	○	○

Q57 **How do you assess the safety of your complementary medicine products?**

Please answer the following questions to help us understand how you assess the safety of the complementary medicine products you use.

Display This Question:

If Q3 = 1

Or Q3 = 3

Q39 I believe a complementary medicine product has been shown to be safe in **pregnancy for the unborn baby** if the following ***health care practitioners*** say it is safe (please choose all that apply)

- ▢ A Naturopath or Herbalist (1)
- ▢ Traditional Chinese (Oriental) Medicine practitioner (2)
- ▢ A Midwife (3)
- ▢ An Integrative Medical Doctor (4)
- ▢ A Child and Family Health Nurse (10)
- ▢ A General Practitioner (GP) (5)
- ▢ An Obstetrician (6)
- ▢ A Pharmacist (7)
- ▢ Pharmacy or health food shop personnel (8)
- ▢ Other (please specify) (9) ____

Display This Question:

If Q3 = 1

Or Q3 = 3

Q41 I believe a complementary medicine product has been shown to be safe in **pregnancy for the unborn baby** if the following ***people in my social circle*** say it is safe (please choose all that apply)

- ▢ Other pregnant women (1)
- ▢ A family member (2)
- ▢ A friend (3)
- ▢ Other (please specify) (5) ____
- ▢ I don't rely on these sources for safety information (4)

Display This Question:

If Q3 = 1

Or Q3 = 3

Q42 I believe a complementary medicine product has been shown to be safe in **pregnancy for the unborn baby** if the following sources say it is safe (please choose all that apply)

- ▢ Hospital or government pamphlets or websites (1)
- ▢ Medication helplines or websites (2)
- ▢ It has been shown to be safe in a research trial (3)
- ▢ Several different internet sites (4)
- ▢ Other (please specify) (6) ____
- ▢ I don't rely on these sources for safety information (5)

Display This Question:

If Q3 = 2

Q44 I believe a complementary medicine product has been shown to be safe in **breastfeeding for the breastfed baby** if the following *health care practitioners* say it is safe (please choose all that apply)

- ▢ A Naturopath or Herbalist (1)
- ▢ Traditional Chinese (Oriental) Medicine practitioner (2)
- ▢ A Midwife (3)
- ▢ An Integrative Medical Doctor (4)
- ▢ A Child and Family Health Nurse (10)
- ▢ A General Practitioner (GP) (5)
- ▢ A Paediatrician (11)
- ▢ An Obstetrician (6)
- ▢ A Pharmacist (7)
- ▢ Pharmacy or health food shop personnel (8)
- ▢ Other (please specify) (9) ____

Display This Question:

If Q3 = 2

Q45 I believe a complementary medicine product has been shown to be safe in **breastfeeding for the breastfed baby** if the following ***people in my social circle*** say it is safe (please choose all that apply)

- ☐ Other breastfeeding women (1)
- ☐ A family member (2)
- ☐ A friend (3)
- ☐ Other (please specify) (5) ____
- ☐ I don't rely on these sources for safety information (4)

Display This Question:

If Q3 = 2

Q46 I believe a complementary medicine product has been shown to be safe in **breastfeeding for the breastfed baby** if the following sources say it is safe (please choose all that apply)

- ☐ Hospital or government pamphlets or websites say it is safe (1)
- ☐ Medication helplines or websites say it is safe (2)
- ☐ It has been shown to be safe in a research trial (3)
- ☐ Several internet sites all say it is safe (4)
- ☐ Other (please specify) (6) ____
- ☐ I don't rely on these sources for safety information (5)

Q47 I believe a complementary medicine product has been shown to be safe **for me** if the following ***health care practitioners*** say it is safe (please choose all that apply)

- ▢ A Naturopath or Herbalist (1)
- ▢ Traditional Chinese (Oriental) Medicine practitioner (2)
- ▢ A Midwife (3)
- ▢ A Child and Family Health Nurse (10)
- ▢ An Integrative Medical Doctor (4)
- ▢ A General Practitioner (GP) (5)
- ▢ An Obstetrician (6)
- ▢ A Pharmacist (7)
- ▢ Pharmacy or health food shop personnel (8)
- ▢ Other (please specify) (9) ____

Q48 I believe a complementary medicine product has been shown to be safe **for me** if the following ***people in my social circle*** say it is (please choose all that apply)

- ▢ Other pregnant women (1)
- ▢ A family member (2)
- ▢ A friend (3)
- ▢ Other (please specify) (4) ____
- ▢ I don't rely on these sources for safety information (5)

Q49 I believe a complementary medicine product has been shown to be safe **for me** if the following ***telephone, online or written sources*** say it is (please choose all that apply)

- ▢ Hospital or government pamphlets or websites (1)
- ▢ Medication helplines or websites (2)
- ▢ It has been shown to be safe in a research trial (3)
- ▢ Several internet sites (4)
- ▢ Other (please specify) (6) ____
- ▢ I don't rely on these sources for safety information (5)

Q52 **The following questions look at the different sources which recommend complementary medicine products (CMPs) and how these may influence whether you would like to take a CMP.**
When considering the complementary medicine products (CMPs) you currently use, how much do you agree with the following statements? There are no right or wrong answers, so please answer as freely as you can.

	Strongly disagree (1)	Disagree (2)	Somewhat disagree (3)	Neither agree nor disagree (4)	Somewhat agree (5)	Agree (6)	Strongly agree (7)
I would take a CMP if recommended by a midwife (1)	o	o	o	o	o	o	o
I would take a CMP if recommended by a GP with additional training in complementary medicines (2)	o	o	o	o	o	o	o
I would take a CMP if recommended by a GP without training in complementary medicines (13)	o	o	o	o	o	o	o
I would take a CMP if recommended by a naturopath or herbalist (3)	o	o	o	o	o	o	o
I would take a CMP if recommended by a Traditional Chinese (Oriental) Medicine practitioner (4)	o	o	o	o	o	o	o

I would take a CMP if recommended by a pharmacist (5)	○	○	○	○	○	○	○
I am not likely to take a CMP unless a health care practitioner recommends it (9)	○	○	○	○	○	○	○
I would take a CMP if recommended by a Child and Family Health Nurse (11)	○	○	○	○	○	○	○
I would take a CMP if recommended by an obstetrician (7)	○	○	○	○	○	○	○
I would take a CMP if recommended by a paediatrician (12)	○	○	○	○	○	○	○
I would take a CMP if recommended by staff in a pharmacy or health food shop (8)	○	○	○	○	○	○	○
Health care professionals have no influence on my decisions to use CMPs (10)	○	○	○	○	○	○	○

Q53 ***When considering the complementary medicine products (CMPs) you currently use, how much do you agree with the following statements? There are no right or wrong answers, so please answer as freely as you can.***

	Strongly disagree (1)	Disagree (2)	Somewhat disagree (3)	Neither agree nor disagree (4)	Somewhat agree (5)	Agree (6)	Strongly agree (7)
I would take a CMP if recommended by other pregnant or breastfeeding women (1)	o	o	o	o	o	o	o
I would take a CMP if recommended by members of my family (2)	o	o	o	o	o	o	o
I would take a CMP if the label says it is for pregnant or breastfeeding women (3)	o	o	o	o	o	o	o
My family members have no influence on my decisions to use CMPs (4)	o	o	o	o	o	o	o

Q58 ***When considering the complementary medicine products you currently use, how much do you agree with the following statements? There are no right or wrong answers, so please answer as freely as you can.***

	Strongly disagree (1)	Disagree (2)	Somewhat disagree (3)	Neither agree nor disagree (4)	Somewhat agree (5)	Agree (6)	Strongly agree (7)
I would take a CMP if recommended in an article I read on the internet (1)	○	○	○	○	○	○	○
I would take a CMP if recommended in a book on pregnancy or breastfeeding health (2)	○	○	○	○	○	○	○
I would take a CMP if recommended in information on a parenting app (3)	○	○	○	○	○	○	○
Information on parenting websites have no influence on my decisions to use CMPs (4)	○	○	○	○	○	○	○
I would take a CMP if recommended in an article I read in a newspaper or magazine (5)	○	○	○	○	○	○	○

Q59 ***When considering the complementary medicine products (CMPs) you currently use, how much do you agree with the following statements? There are no right or wrong answers, so please answer as freely as you can.***

	Strongly disagree (1)	Disagree (2)	Somewhat disagree (3)	Neither agree nor disagree (4)	Somewhat agree (5)	Agree (6)	Strongly agree (7)
I would take a CMP if recommended in pamphlets or other information from women's hospitals (1)	o	o	o	o	o	o	o
I would take a CMP if recommended on government-run websites (2)	o	o	o	o	o	o	o
I would take a CMP if recommended on hospital medication websites like MotherSafe (3)	o	o	o	o	o	o	o
I would only take a CMP after I have received information from a health care professional and read up on it (4)	o	o	o	o	o	o	o
I would only take a CMP after I have read up on it from trusted sources of information (5)	o	o	o	o	o	o	o

When thinking about taking a CMP, I am more likely to take it if I find similar information from several sources (6)	○	○	○	○	○	○	○
When thinking about taking a CMP, I prefer to look at information from several sources about the CMP before I will consider taking it (7)	○	○	○	○	○	○	○

Q60

Your reasons for taking complementary medicine products
The following questions explore your motivations for using complementary medicine products

---

Display This Question:

If Q3 = 1

Or Q3 = 3

Q61

Please think about the complementary medicine products (CMPs) you currently take in **pregnancy.** On a scale of 1-10, how much do you agree with the following statements?

	Do not agree at all	Neither agree nor disagree	Completely agree
	1 2 3 4	5 6 6 7	8 9 10
I take CMPs to benefit my unborn baby ()			
I take CMPs for the benefit of my own health ()			
I take CMPs for the benefit of my pregnancy ()			
I only take CMPs after considering possible harms to my baby ()			

Page Break

Display This Question:

If Q3 = 2

Q62
Please think about the complementary medicine products (CMPs) you currently take during **breastfeeding.** On a scale of 1-10, how much do you agree with the following statements?

	Completely disagree	Neither agree nor disagree	Completely agree
	1 2 3 4	5 6 6 7	8 9 10
I take CMPs to benefit my breastfeeding baby ()			
I take CMPs for the benefit of my own health ()			
I take CMPs to help me breastfeed successfully ()			
I take CMPs to increase my breastmilk supply ()			
I only take CMPs after considering possible harms to my breastfeeding baby ()			
I only take CMPs after considering possible harms to my breastmilk supply ()			

Q66
Your previous use of complementary medicine products

We would like to know about women's experiences of using complementary medicine products. The next questions are about any complementary medicine products you may have
used in the past, both before and during pregnancy or breastfeeding.

When thinking about the complementary medicine products you have used in the past and during pregnancy, please answer the following questions

---

Q68 I have taken CMPs before without any issues

- Yes (1)
- No (2)
- I don't know (3)

Q69 I have previously used CMPs and they have been beneficial to my own health

- Yes (1)
- No (2)
- I don't know (3)

Q70 I have previously used CMPs and they have been beneficial to my pregnancy

- Yes (1)
- No (2)
- I don't know (3)

Q71 I have previously used CMPs and they have been beneficial to my unborn baby's heath

- Yes (1)
- No (2)
- I don't know (3)

Display This Question:

If Q3 = 2

Q72 I have previously used CMPs and they have been beneficial to my breastfeeding baby's heath

- Yes (1)
- No (2)
- I don't know (3)

Q73 When thinking about your preferred health care choices in general, please indicate how much you agree or disagree with the following statements.

	Strongly disagree (1)	Disagree (2)	Somewhat disagree (3)	Neither agree nor disagree (4)	Somewhat agree (5)	Agree (6)	Strongly agree (7)
Whenever possible I prefer to use complementary medicine to maintain my health (1)	o	o	o	o	o	o	o
I prefer to use complementary medicine products to prevent ill health (2)	o	o	o	o	o	o	o
Whenever possible I prefer to use complementary medicine products rather than pharmaceutical medications (3)	o	o	o	o	o	o	o
When I am ill, I prefer to use medicines prescribed by my pharmacist or GP (4)	o	o	o	o	o	o	o
When I am ill, I prefer to use medicines prescribed by my most trusted health care practitioner (5)	o	o	o	o	o	o	o

When I am ill, I prefer to use medicines prescribed by a naturopath or herbalist (6)	○	○	○	○	○	○	○
When I am ill, I prefer to use medicines prescribed by a Traditional Chinese (Oriental) Medicine practitioner (7)	○	○	○	○	○	○	○
When I am ill, I prefer to use complementary medicine products to treat my condition (8)	○	○	○	○	○	○	○

End of Block: Block 1

Start of Block: Default Question Block

Q65 Please consider your beliefs around taking CMPs and indicate how much you agree or disagree with the following statements

Display This Question:

If Q3 = 1

Or Q3 = 3

Q63 **The following questions are about your beliefs regarding taking complementary medicine products (CMPs) during pregnancy.**

Please consider your beliefs around taking CMPs and indicate how much you agree or disagree with the following statements

	Strongly disagree (1)	Disagree (2)	Somewhat disagree (3)	Neither agree nor disagree (4)	Somewhat agree (5)	Agree (6)	Strongly agree (7)
I believe that it is important that I have a healthy baby, therefore I take CMPs during pregnancy (1)	o	o	o	o	o	o	o
I believe that it is important that I stay as healthy as possible as a pregnant mother, therefore I take complementary medicine products (2)	o	o	o	o	o	o	o

Display This Question:

If Q3 = 2

Q64 **The following questions are about your beliefs regarding taking complementary medicine products (CMPs) during breastfeeding.**

Please consider your beliefs around taking CMPs and indicate how much you agree or disagree with the following statements

Q40 Please tell us the other things that show you a complementary medicine product is safe for the **unborn baby**

____

Q18 What other information source did you use to get information on your supplements?

- ◯ No other information source (1)
- ◯ I get information on my dietary supplements from (please specify) (2) ____

Q50 Please tell us the other things that show you a complementary medicine product is safe for **you**

____

Q43 Please tell us the other things that show you a complementary medicine product is safe for the **breastfeeding baby**

____

Q51 I believe a complementary medicine product has been shown to be safe **for me** if the following ***people in my social circle*** such as (please choose all that apply)

- ▢ Other breastfeeding women have used it without side effects (1)
- ▢ A family member says it is safe (2)
- ▢ A friend says it is safe (3)

Q68 I have taken CMPs before without any issues

- ◯ Yes (1)
- ◯ No (2)

Q12 **We would like to understand the dietary supplements women use in pregnancy, or when breastfeeding, and why. Examples of dietary supplements include multivitamins, vitamin or mineral supplements (e.g. iron tablets) or probiotics.**

Please tell us about the dietary supplements you currently use

---

Display This Question:

If Q3 = 1

Or Q3 = 3

Q77 **Personal beliefs about a healthy pregnancy.**
Each item below is a brief statement about your health with which you may agree or disagree. This is a measure of your personal beliefs, so there are no right or wrong answers.

Display This Question:

If Q3 = 1

Or Q3 = 3

Q79 Please consider your overall **health and well-being during your pregnancy**, and imagining if your health was poor or very good, choose how strongly you agree or disagree with the following statements.

	Strongly disagree (1)	Moderately disagree (2)	Slightly disagree (3)	Slightly agree (4)	Moderately agree (5)	Strongly agree (6)
If my health and well-being during pregnancy worsens, it is my own behaviour which determines how soon I will feel better again (1)	o	o	o	o	o	o
As to my health and well-being during pregnancy, what will be will be (2)	o	o	o	o	o	o
If I see my health care practitioner (e.g. midwife, doctor, naturopath or other) regularly, I am less likely to have problems with my health and well-being during pregnancy (3)	o	o	o	o	o	o
Most things that affect my health and well-being during pregnancy happen to me by chance (4)	o	o	o	o	o	o

Whenever my health and well-being during pregnancy worsens, I should consult a health care practitioner (e.g. doctor, midwife, naturopath or other) (5)	○	○	○	○	○	○
I am directly responsible for my health and well-being during pregnancy getting better or worse (6)	○	○	○	○	○	○
Other people play a big role in whether my health and well-being during pregnancy improves, stays the same or gets worse (7)	○	○	○	○	○	○
Whatever goes wrong with my health and well-being during pregnancy is my own fault (8)	○	○	○	○	○	○
Luck plays a big part in determining how my health and well-being during pregnancy improves (9)	○	○	○	○	○	○
In order for my health and well-being during pregnancy to improve, it is up to other people to see that the right things happen (10)	○	○	○	○	○	○

Whatever improvement occurs with my health and well-being during pregnancy is largely a matter of good fortune (11)	○	○	○	○	○	○
The main thing which affects my health and well-being during pregnancy is what I myself do (12)	○	○	○	○	○	○
I deserve the credit when my health and well-being during pregnancy improves and the blame when it gets worse (13)	○	○	○	○	○	○
Following my health care practitioner's (e.g. my doctor's, midwife's, naturopath's or other's) orders to the letter is the best way to keep my health and well-being during pregnancy from getting any worse (14)	○	○	○	○	○	○
If my health and well-being during pregnancy worsens, it's a matter of fate (15)	○	○	○	○	○	○
If I am lucky, my health and well-being during pregnancy will get better (16)	○	○	○	○	○	○

If my health and well-being during pregnancy takes a turn for the worse, it is because I have not been taking proper care of myself (17)	o	o	o	o	o	o
The type of help I receive from other people determines how soon my pregnancy health improves (18)	o	o	o	o	o	o

---

Display This Question:

If Q3 = 2

Q81 **Personal beliefs about healthy breastfeeding.**
Each item below is a brief statement about your health with which you may agree or disagree. This is a measure of your personal beliefs, so there are no right or wrong answers.

Display This Question:

If Q3 = 2

Q83 Please consider your overall **health and well-being as a breastfeeding mother**, imagining if your health was poor or very good, and choose how strongly you agree or disagree with the following statements.

	Strongly disagree (1)	Moderately disagree (2)	Slightly disagree (3)	Slightly agree (4)	Moderately agree (5)	Strongly agree (6)
If my health and well-being as a breastfeeding mother worsens, it is my own behaviour which determines how soon I will feel better again (1)	○	○	○	○	○	○
As to my health and well-being as a breastfeeding mother, what will be will be (2)	○	○	○	○	○	○
If I see my health care practitioner (e.g. midwife, doctor, naturopath or other) regularly, I am less likely to have problems with my health and well-being as a breastfeeding mother (3)	○	○	○	○	○	○
Most things that affect my health and well-being as a breastfeeding mother happen to me by chance (4)	○	○	○	○	○	○

Whenever my health and well-being as a breastfeeding mother worsens, I should consult a health care practitioner (e.g. doctor, midwife, naturopath or other) (5)	○	○	○	○	○	○
I am directly responsible for my health and well-being as a breastfeeding mother getting better or worse (6)	○	○	○	○	○	○
Other people play a big role in whether my health and well-being as a breastfeeding mother stays the same or gets worse (7)	○	○	○	○	○	○
Whatever goes wrong with my health and well-being as a breastfeeding mother is my own fault (8)	○	○	○	○	○	○
Luck plays a big part in determining how my health and well-being as a breastfeeding mother improves (9)	○	○	○	○	○	○
In order for my health and well-being as a breastfeeding mother to improve, it is up to other people to see that the right things happen (10)	○	○	○	○	○	○

Whatever improvement occurs with my health and well-being as a breastfeeding mother is largely a matter of good fortune (11)	○	○	○	○	○	○
The main thing which affects my health and well-being as a breastfeeding mother is what I myself do (12)	○	○	○	○	○	○
I deserve the credit when my health and well-being as a breastfeeding mother improves and the blame when it gets worse (13)	○	○	○	○	○	○
Following my health care practitioner's (e.g. my doctor's, midwife's, naturopath's or other's) orders to the letter is the best way to keep my health and well-being as a breastfeeding mother from getting any worse (14)	○	○	○	○	○	○
If my health and well-being as a breastfeeding mother worsens, it's a matter of fate (15)	○	○	○	○	○	○

If I am lucky, my health and well-being as a breastfeeding mother will get better (16)	○	○	○	○	○	○
If my health and well-being as a breastfeeding mother takes a turn for the worse, it is because I have not been taking proper care of myself (17)	○	○	○	○	○	○
The type of help I receive from other people determines how soon my health and well-being as a breastfeeding mother improves (18)	○	○	○	○	○	○

End of Block: Block 4

Start of Block: Default Question Block

Q92 The questions in this short section of the survey will help us understand how well you understand health information and can interpret it to make health decisions

---

Q93 **How confident are you filling out medical forms by yourself?**

- ○ Extremely (1)
- ○ Quite a bit (2)
- ○ Somewhat (3)
- ○ A little bit (4)
- ○ Not at all (5)

Q94 Please read the nutritional panel below to answer the following questions. The panel is information on the back of a container of ice cream.

Product description: Ice Cream

Serving size	100ml
Servings per container	4

NUTRITIONAL INFORMATION

TYPICAL VALUES	*Per 100ml*
**Energy**	**1050 kJ**
	**250 kcal (calories)**
**Protein**	**4 g**
**Carbohydrate**	**30 g**
of which sugars	23 g
**Fat**	**13 g**
of which saturates	9 g
of which monounsaturates	0 g
of which polyunsaturates	3 g
of which trans fats	1 g
**Fibre**	**0 g**
**Sodium**	**0.05 g**

Ingredients: Cream, Skimmed Milk, Sugar, Whole Egg, Stabilisers (Guar Gum), Peanut Oil, Vanilla Extract (0.05%)

---

*

Q95 How many calories (kcal) will you eat if you eat the whole container?

____

Q96 If you are advised to eat no more than 60 grams of carbohydrates for dessert, what is the maximum amount of ice cream you could have?

	Amount	Unit of measurement
	(Use a number. Half can be written as '0.5') (1)	
Ice cream (1)		▼ ml (1 ... serve(s) (3)

Q97 Imagine that your doctor advises you to reduce the amount of saturated fat in your diet. You usually have 42 g of saturated fat each day, some of which comes from one serving of ice cream. If you stop eating ice cream, how many grams of saturated fat would you be eating each day?

○ g (grams) (1) ____

Q99 If you usually eat 2,500 calories in a day, what percentage of your daily value of calories will you be eating if you eat one serving?

○ % (per cent) (1) ____

Q100 Pretend that you are allergic to the following substances: penicillin, peanuts, latex gloves, and bee stings.
Is it safe for you to eat this ice cream?

○ Yes (1)

○ No (2)

Display This Question:

If Q100 = 2

Q101 Why not?

____

Q102 **About You**

The following questions tell us about the people who have completed the survey. Please consider completing this section.

Q103 I am currently

- ○ Single (1)
- ○ Married or in a de facto relationship (2)
- ○ Separated or divorced (3)
- ○ Widowed (4)
- ○ Other (please specify) (5) ____

---

*

Q104 What year were your born in?
Please enter the year of your birth in numerals, e.g. 1990

____

Q105 Please choose your highest level of education from the list below

- ○ Primary school (1)
- ○ Year 10 (School certificate) (2)
- ○ Year 12 (high school certificate) or equivalent (3)
- ○ Certificate 1-4 (4)
- ○ Diploma (5)
- ○ Associate Diploma (6)
- ○ Bachelor Degree (7)
- ○ Postgraduate studies at university (8)
- ○ Other (please specify) (9) ____

Q106 What is your approximate household income per week?

- No income (1)
- $1 - $456 per week (2)
- $457 - $960 per week (3)
- $961 - $1616 per week (4)
- $1617 - $2489 per week (5)
- $2490 - $5036 per week (6)
- $5037 or above per week (7)
- I prefer not to answer (8)

Q107 What country were you born in?

- Australia (1)
- The United Kingdom (2)
- New Zealand (3)
- China (4)
- India (5)
- The Philippines (6)
- Vietnam (7)
- Italy (8)
- South Africa (9)
- Malaysia (10)
- Germany (11)
- Other (please tell us the country you were born in) (12) __________

Q108 What country was your mother born in?

- ◯ Australia (1)
- ◯ The United Kingdom (2)
- ◯ New Zealand (3)
- ◯ China (4)
- ◯ India (5)
- ◯ The Philippines (6)
- ◯ Vietnam (7)
- ◯ Italy (8)
- ◯ South Africa (9)
- ◯ Malaysia (10)
- ◯ Germany (11)
- ◯ Other (please tell us the country your mother was born in) (12) __________

Q109 What country was your father born in?

- ○ Australia (1)
- ○ The United Kingdom (2)
- ○ New Zealand (3)
- ○ China (4)
- ○ India (5)
- ○ The Philippines (6)
- ○ Vietnam (7)
- ○ Italy (8)
- ○ South Africa (9)
- ○ Malaysia (10)
- ○ Germany (11)
- ○ Other (please tell us the country your father was born in) (12) ________________

Q110 What main language do you speak at home?

- ○ English (1)
- ○ Italian (2)
- ○ Greek (3)
- ○ Cantonese (4)
- ○ Arabic (5)
- ○ Mandarin (6)
- ○ Vietnamese (7)
- ○ Malay (8)
- ○ German (9)
- ○ Tagalog (10)
- ○ Other (please tell us the main language you speak at home) (11) ________________

Q111 Do you currently smoke?

- ○ Yes (1)
- ○ No (2)
- ○ Prefer not to respond (3)

If you have any questions or concerns regarding the complementary medicines you are using in pregnancy or breastfeeding, please discuss these with your health care practitioner. Additionally, the following resources may be able to provide you with some information on breastfeeding or pregnancy concerns.

Complementary medicine use in pregnancy or breastfeeding
The Royal Women's Hospital in Melbourne, telephone (03) 8345 2000 or (03) 9076 1000. For additional information on complementary medicines in pregnancy and lactation please see these links:
Food and nutrition in pregnancy:
https://www.thewomens.org.au/health-information/pregnancy-and-birth/a-healthy-pregnancy/food-nutrition-in-pregnancy

Herbal and traditional medicines in pregnancy:
https://thewomens.r.worldssl.net/images/uploads/fact-sheets/Herbal-traditional-medicines-in-pregnancy.pdf

Herbal and traditional medicines in breastfeeding:
https://thewomens.r.worldssl.net/images/uploads/fact-sheets/Herbal-medicines-in-pregnancy-breastfeeding-171018.pdf

Women's health information fact sheets A-Z from the Royal Women's Hospital, Melbourne:
https://www.thewomens.org.au/health-information/fact-sheets

NSW participants can access **MotherSafe** at the Royal Hospital for Women 02 9382 6539 or 1800 647 848 (NSW country) for information on medications taken in pregnancy and breastfeeding.
Participants from all Australian States and Territories can access the **NPS Medicines Line** (9am-5pm) 1300 633 424 for information on medications taken in pregnancy and breastfeeding.

Poisons information (available 24 hours / day, 7 days a week) 13 11 26 (**all Australian States and Territories**)

Breastfeeding specific queries

The **Australian Breastfeeding Association** helpline number 1800 686 268 for help with breastfeeding and parenting support (available 24 hours / day, 7 days a week). Their website also has a lot of breastfeeding, mothering and parent support: https://www.breastfeeding.asn.au/

Lactation Consultants of Australia and New Zealand http://www.lcanz.org/ for help finding a qualified lactation consultant.
