# Supplementary material for: Demographics, health literacy and health locus of control beliefs of Australian women who take complementary medicine products during pregnancy and breastfeeding: A cross‐sectional, online, national survey
Source: Health Expect. 2021 Dec 23;25(2):667–83. doi: 10.1111/hex.13414 (PMC8957740; doi:10.1111/hex.13414)
Supplement: Supplementary file 2 — Supporting information. [file HEX-25--s003.docx]

Completed Checklist for Reporting Results of Internet E‐Surveys (CHERRIES) [1, 2]

| ***Checklist Item*** | ***Explanation*** | ***Sections*** |
| --- | --- | --- |
| 1. Describe survey design | ***Describe target population, sample frame. Is the sample a convenience sample? (In “open” surveys this is most likely.)***   - Target population: Participants were women over 18 years of age who were currently pregnant and/or breastfeeding, taking complementary medicine products (CMPs), and living in Australia. - Participants also needed to have access to the Internet and be proficient in English to complete the questionnaire. - Although the survey was an ‘open survey’ (see item 6 below) and participants voluntarily self‐ selected to participate, some principles of statistical research were used to calculate approximate sample sizes for the two subgroups included (pregnant women and breastfeeding women). | Methods section  *Survey design*  *Sample size calculations* |
| 2. IRB approval | ***Mention whether the study has been approved by an IRB.***   - Ethical approval for the study was obtained from The University of Sydney Human Research Ethics Committee (approval number 2018/1010). | Methods section  *Ethical considerations* |
| 3. Informed  consent | ***Describe the informed consent process. Where were the participants told the length of time of the survey, which data were stored and where and for how long, who the investigator was, and the purpose of the study?***  A comprehensive Participant Information Statement (PIS) appeared before any survey questions were presented, and informed participants of the following:   - that completion of the questionnaire was taken as consent to participate. - that it would take approximately twenty minutes to complete the questionnaire. - that survey data would be collected at the Qualtrics secure data centre in Sydney NSW, and thereafter exported data would be stored securely in password protected files on secure networks at the University of Sydney and will only be accessed by researchers directly involved in   the study. | Declarations section  *Ethics approval and consent to participate* |

|  | - that the survey was anonymous and no identifying data (including IP addresses) would be collected except postcode and pregnancy or breastfeeding status - the names of the research team members, their roles in the research, and email addresses and telephone numbers and email addressed for the Chief Investigator (PA) and Student Investigator (LAJB). - Telephone and email address for the Manager, Ethics Administration at The University of Sydney (if participants had any concerns and wished to make a complaint).   Although not included on the PIS, the data will be stored for 5 years as per legislative and University of Sydney requirements and the Sydney University Human research Ethics Committee approved protocol. |  |
| --- | --- | --- |
| 4. Data protection | ***If any personal information was collected or stored, describe what mechanisms were used to protect unauthorized access.***  During data collection, the data in Qualtrics was stored on Australian soil. Access to the data in Qualtrics was protected through security controls implemented by Qualtrics. This involved Access control and Authorisation. Key points from the Qualtrics security paper[3] are copied below:  *“Access Control:*   - Passwords are stored in Qualtrics using one‐way, salted encryption - No remote software is installed on general workstations - Secured login, passwords are encrypted, automated log‐out   *Authorization:*   - Authentication is using secure HTTP (TLSv1.2). The customer’s web browser must support 128‐bit or higher TLS protocol encryption. - Authentication can be extended to integrate with customer’s single sign‐on (SSO): LDAP, CAS, OAuth, SAML, Token, or Shibboleth.” - After data collection finished, Electronic data was exported from Qualtrics and stored in the Research Data Store at The University of Sydney. The data remains password protected, and the Masterfile with possible identifying data (postcode and pregnancy or breastfeeding status) was encrypted using VeraCrypt. - Responses to the survey were anonymous. However, at the end of the survey, participants were given the option of entering their email addresses to go in the draw to win an iPad mini | Not stated in the manuscript, but detailed here, and in the associated Methods paper [4] |

|  | and/or to receive a summary of the overall results of the study. If they chose either of these options, they were automatically redirected to a separate survey so that their email addresses were not linked to the information gathered in the study survey. After the draw and summary of results were distributed, email address lists were destroyed. |  |
| --- | --- | --- |
| 5. Development and testing | ***State how the survey was developed, including whether the usability and technical functionality of the electronic questionnaire had been tested before fielding the questionnaire.***   - A pilot questionnaire was designed by the research team, all of whom have experience of pregnancy and motherhood, and three of whom have clinical experience of working with pregnant or breastfeeding women as a naturopath (LAJB), pharmacist (PA) and midwife (LB) respectively. Content validity was assessed through consensus and discussion by the research team, and face and content validity were confirmed through piloting the survey with volunteers. - Piloting of the questionnaire on the Qualtrics platform with volunteers also informed the ease and usability of the survey tool. It took between 17 and 25 minutes for each of the volunteers to complete the questionnaire. Volunteers completed the questionnaire on tablets, mobile telephones and laptops, and trialled the questionnaire twice each (once as a pregnant or pregnant and breastfeeding participant, and once as a breastfeeding only participant). Feedback was generally positive. The volunteers stated that the questionnaire flowed well, made sense and covered areas they expected in a survey on complementary medicine product use in pregnancy and breastfeeding. They did not suggest any wording changes to the questions, nor did they suggest any additional questions. Furthermore, their understanding of the questions and purpose of the study was aligned with our understanding. Thus, we confirmed the face and   content validity of the survey items. | Methods section  *Survey design* |
| 6. Open survey versus closed survey | ***An “open survey” is a survey open for each visitor of a site, while a closed survey is only open to a sample which the investigator knows (password‐protected survey).***   - Open survey | Not applicable |
| 7. Contact mode | ***Indicate whether or not the initial contact with the potential participants was made on the Internet. (Investigators may also send out questionnaires by mail and allow for Web‐based data entry.)***   - Initial contact with all potential participants was made on the Internet. | Methods section  *Recruitment* |

| 8. Advertising the survey | ***How/where was the survey announced or advertised? Some examples are offline media (newspapers), or online (mailing lists – If yes, which ones?) or banner ads (Where were these banner ads posted and what did they look like?). It is important to know the wording of the announcement as it will heavily influence who chooses to participate. Ideally the survey announcement should be published as an appendix.***   - Participants were recruited to the survey entirely online, primarily through a Facebook page specific to the research project and paid promoted posts generated from this Facebook page. - Additionally, the survey was advertised on the free online advertising platform Gumtree and through the electronic communication platform Yammer at The University of Sydney [4]. | Methods section  *Recruitment* |
| --- | --- | --- |
| 9. Web/E‐mail | ***State the type of e‐survey (eg, one posted on a Web site, or one sent out through e‐mail). If it is an e‐mail survey, were the responses entered manually into a database, or was there an automatic method for capturing responses?***   - The survey was hosted on the online Qualtrics platform. Participants accessed it through a link included in Facebook posts, the Gumtree advertisement and Yammer. - The Qualtrics platform enabled all responses to be collected on the online database directly as participants answered the questionnaire. No manual data entry was necessary. | Methods section  *Survey design* |
| 10. Context | ***Describe the Web site (for mailing list/newsgroup) in which the survey was posted. What is the Web site about, who is visiting it, what are visitors normally looking for? Discuss to what degree the content of the Web site could pre‐select the sample or influence the results. For example, a survey about vaccination on an anti‐immunization Web site will have different results from a Web survey conducted on a government Web site***   - Not applicable as the survey was not distributed through mailing lists of newgroups. | Not applicable |
| 11. Mandatory / voluntary | ***Was it a mandatory survey to be filled in by every visitor who wanted to enter the Web site, or was it a voluntary survey?***   - Participation was completely voluntary, as indicated in the Participant Information Statement (PIS) that appeared as the first page of the survey. The statement included the information that completion of the questionnaire was taken as consent to participate. | Declarations section  *Ethics approval and consent to participate* |
| 12. Incentives | ***Were any incentives offered (eg, monetary, prizes, or non‐monetary incentives such as an offer to provide the survey results)?*** | Declarations section |


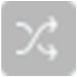


|  | - At the end of the survey, participants were given the option of entering their email addresses to go in the draw to win an iPad mini and/or to receive a summary of the overall results of the study. If they chose either of these options, they were automatically redirected to a separate survey so that their email addresses were not linked to the information gathered in the study   survey. | *Ethics approval and consent to participate* |
| --- | --- | --- |
| 13. Time / Date | ***In what timeframe were the data collected?***   - Recruitment and data collection occurred from July 17th to September 17th, 2019. | Methods section  *Recruitment* |
| 14. Randomization of items or questionnaires | ***To prevent biases items can be randomized or alternated.***   - The Qualtrics platform enabled items to be randomised within individual survey items. Randomisation was included wherever practicable to reduce response order bias. For example, for Question 8 (pasted below) the options were randomised and appeared in a different order each time the survey was accessed by a participant.   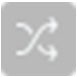  “Q8 **You and your chosen health care practitioners.**  As a **pregnant mother**, which one of the following health care practitioners do you **most trust** when seeking health care for **yourself** during pregnancy?   - Naturopath or herbalist - Midwife - Integrative medical doctor - Traditional Chinese (Oriental) Medicine practitioner - Child and Family Health Nurse - General Practitioner (GP) - Obstetrician - Pharmacist - Other (please specify)” | Additional File 1  *This is not detailed in the manuscript.*  *However, Additional File 1 contains the complete survey.* |
|  |  | *Where this symbol appears, response options were randomised, such as in the example presented here.* |

| 15. Adaptive  questioning | ***Use adaptive questioning (certain items, or only conditionally displayed based on responses to other items) to reduce number and complexity of the questions.***The questionnaire contained a mixture of items that all participants were asked to complete, some items only pertinent to pregnant participants, and some items only pertinent to breastfeeding participants. The questionnaire was structured so that questions that were only pertinent to pregnant participants did not appear for breastfeeding participants and vice versa.  Where questions only appeared for ***pregnant participants only*** the following display conditions can be seen in Additional File 1.  *Display This Question: If Q3 = 1*  *Or Q3 = 3*  Q3=1 was chosen by women who were currently pregnant; Q3=3 was chosen by women who were both currently pregnant and breastfeeding.  Where questions appeared for ***breastfeeding participants only*** the follow display condition can be seen in Additional File 1.  *Display This Question: If Q3 = 2*  Q3=2 was chosen by women who were currently breastfeeding, but not pregnant.  Additionally, some survey items conditionally appeared depending on answers to a previous question. For example, in Question 20 participants were asked what herbal medicines they took. If they chose the option *‘I do not currently take any herbal medicines’* than the subsequent questions  inquiring about herbal medicines information and recommendation sources did not appear. | Additional File 1  *This is not detailed in the manuscript.*  *However, Additional File 1 contains the complete survey, with the display options clearly marked.* |
| --- | --- | --- |
| 16. Number of Items | ***What was the number of questionnaire items per page? The number of items is an important factor for the completion rate.***   - Depending on the number of options a participant could choose in each questionnaire item, between 1 and 4 questions appeared per page. For example, for Question 20 (pasted below), a comprehensive checklist was provided for participants, and this was the only question that | Not detailed in the manuscript as not applicable to |

|  | appeared on the page. For questions with fewer response items, up to four questions appeared | the results |
| --- | --- | --- |
|  | on the page. | presented. |
|  | *Note: the complete survey appears in Appendix K.* |  |
|  | Q20 I currently take the following types of herbal medicines. (Please choose all that apply) |  |
|  | - Raspberry leaf - Cranberry - Echinacea - Ginger - Peppermint - Chamomile - Fenugreek - Fennel - St Mary's thistle (Milk thistle) - Dong quai - Shatavari - Herbal extracts or teas from my health practitioner - Other (please specify) - I do not currently take any herbal medicines |  |
| 17. Number of | ***Over how many pages was the questionnaire distributed? The number of items is an important factor for the completion rate.***   - The questionnaire was distributed over 16 pages. - The page breaks enabled the survey to appear clearly on both small screen sizes (like smart phones) and larger screens (home computers, laptops and tablets) is recommended on the Qualtrics platform to reduce survey fatigue. - Additionally, the questionnaire included a ‘progress bar’ at the top of each page showing the percentage of the survey completed so that participants could track their progress as they participated. | Not detailed in |
| screens (pages) |  | the manuscript |
|  |  | as not |
|  |  | applicable to |
|  |  | the results |
|  |  | presented. |
| 18. Completeness check | ***It is technically possible to do consistency or completeness checks before the questionnaire is submitted. Was this done, and if “yes”, how (usually JAVAScript)? An alternative is to check for completeness after the questionnaire has been submitted (and highlight mandatory items). If this*** | Not detailed in the manuscript as not |

|  | ***has been done, it should be reported. All items should provide a non‐response option such as “not applicable” or “rather not say”, and selection of one response option should be enforced.***   - Partially completed surveys were held in the Qualtrics platform as *‘Responses in progress’* for ten days before being submitted to the completed *‘Recorded responses’* data storage for finalised surveys. - The *‘Recorded responses’* data included two variables: finished (true/false) and indicated if the participant had progressed through the questionnaire to the final question; and ‘*Progress’* which indicated the percentage of the questionnaire that was completed. - Only three compulsory screening questions (Questions 1, 4 and 5 – pasted below) were included in the questionnaire. These appeared at the beginning of the questionnaire and ensured participants met the inclusion criteria for participation. They assessed whether participants were currently pregnant and/or breastfeeding; currently used complementary medicine products; and currently lived in Australia.   Q1 Are you currently   - - Pregnant   - Breastfeeding   - Both pregnant and breastfeeding   - Neither pregnant or breastfeeding 🡪 if this answer was chosen, the participant was redirected to a message saying ‘*Thank you for your interest. Unfortunately, you need to be pregnant and / or breastfeeding to complete this survey’* and then exited the survey   Q4 Do you currently take any complementary medicine products?  *CMPs are defined as the following:*   - *Products like herbal medicines (e.g. in teas, tablets, capsules or extracts), or vitamin and mineral supplements (e.g. multivitamins, iodine supplements, fish oils or probiotics).* - *Some vitamins and minerals have a scientific evidence base (e.g. iron, folic acid or iodine supplements), and may be recommended by your doctor or other healthcare practitioner.* - *Some CMPs like some herbal medicines may have traditional uses but may not have been scientifically researched.* - Yes - No 🡪 if this answer was chosen, the participant was redirected to a message saying ‘*Thank*   *you for your interest in the study. Unfortunately you need to be currently taking a* | applicable to the results presented. |
| --- | --- | --- |

|  | *complementary medicine product like herbal medicines or vitamin supplements to complete the survey’* and then exited the survey  Q5 Do you live in Australia?   - Yes - No 🡪 if this answer was chosen, the participant was redirected to a message saying *‘Thank you for your interest in the study. Unfortunately you need to be an Australian resident to*   *complete the survey*’ and then exited the survey |  |
| --- | --- | --- |
| 19. Review step | ***State whether respondents were able to review and change their answers (eg, through a Back button or a Review step which displays a summary of the responses and asks the respondents if they are correct).***   - A review step was not included. | Not applicable |
| 20. Unique site visitor | ***If you provide view rates or participation rates, you need to define how you determined a unique visitor. There are different techniques available, based on IP addresses or cookies or both.***   - We are unable to compute this as participants’ IP addresses were not recorded to help ensure anonymity. However, the Qualtrics program did inject cookies and cache into the browser a participant would use to access the survey. This enabled the participant to exit the survey and re‐enter at the point at which she had previously exited, and helped prevent duplicate entries. - *There were 1444 recorded responses in total including 26 pilot or trial responses; for the final analysis 1418 were included in the completion rate calculation (item 23 below).* | Not detailed in the manuscript.  Results section, *Responses collected* |
| 21. View rate (Ratio of unique survey visitors/unique site visitors) | ***Requires counting unique visitors to the first page of the survey, divided by the number of unique site visitors (not page views!). It is not unusual to have view rates of less than 0.1 % if the survey is voluntary.***   - Because participants’ IP addresses were not recorded, this cannot be determined. | Not applicable |
| 22. Participation rate (Ratio of unique visitors who agreed to  participate/uniq | ***Count the unique number of people who filled in the first survey page (or agreed to participate, for example by checking a checkbox), divided by visitors who visit the first page of the survey (or the informed consents page, if present). This can also be called “recruitment” rate.***   - Not applicable | Not applicable |

| ue first survey page visitors) |  |  |
| --- | --- | --- |
| 23. Completion rate (Ratio of users who finished the survey/users who agreed to participate) | ***The number of people submitting the last questionnaire page, divided by the number of people who agreed to participate (or submitted the first survey page). This is only relevant if there is a separate “informed consent” page or if the survey goes over several pages. This is a measure for attrition. Note that “completion” can involve leaving questionnaire items blank. This is not a measure for how completely questionnaires were filled in. (If you need a measure for this, use the word “completeness rate”.)***   - 810 participants submitted the last questionnaire page and 1418 participants agreed to participate (as indicated by completing the first survey page) - 810/1418 = 57.1% | Results section  *Responses collected* |
| 24. Cookies used | ***Indicate whether cookies were used to assign a unique user identifier to each client computer. If so, mention the page on which the cookie was set and read, and how long the cookie was valid. Were duplicate entries avoided by preventing users access to the survey twice; or were duplicate database entries having the same user ID eliminated before analysis? In the latter case, which entries were kept for analysis (eg, the first entry or the most recent)?***   - Cookies were used to assign a unique user identifier to each client computer. These cookies were set on the public survey link generated from the Qualtrics platform and remained in place for 1 week only. - This gave participants a week to re‐enter and complete the survey if they had had to leave prior to completing it, as described in item 20 above. | Not detailed in the manuscript as not applicable to the results presented. |
| 25. IP check | ***Indicate whether the IP address of the client computer was used to identify potential duplicate entries from the same user. If so, mention the period of time for which no two entries from the same IP address were allowed (eg, 24 hours). Were duplicate entries avoided by preventing users with the same IP address access to the survey twice; or were duplicate database entries having the same IP address within a given period of time eliminated before analysis? If the latter, which entries were kept for analysis (eg, the first entry or the most recent)?***   - Not applicable – participants’ IP addresses were not recorded. | Not applicable |
| 26. Log file analysis | ***Indicate whether other techniques to analyze the log file for identification of multiple entries were used. If so, please describe.*** | Not applicable |

|  | - Not applicable – participants’ IP addresses were not recorded. |  |
| --- | --- | --- |
| 27. Registration | ***In “closed” (non‐open) surveys, users need to login first and it is easier to prevent duplicate entries from the same user. Describe how this was done. For example, was the survey never displayed a second time once the user had filled it in, or was the username stored together with the survey results and later eliminated? If the latter, which entries were kept for analysis (eg, the first entry or the most recent)?***   - Not applicable | Not applicable |
| 28. Handling of incomplete questionnaires | ***Were only completed questionnaires analyzed? Were questionnaires which terminated early (where, for example, users did not go through all questionnaire pages) also analyzed?***   - Only completed questionnaires were analysed, i.e. those questionnaires where participants went through all questionnaire pages to the end of the survey. Note, because all questions except the screening questions were voluntary, missing responses have been reported in the results after data analysis. | Results section  *Responses collected* |
| 29. Questionnaires submitted with an atypical timestamp | ***Some investigators may measure the time people needed to fill in a questionnaire and exclude questionnaires that were submitted too soon. Specify the timeframe that was used as a cut‐off point, and describe how this point was determined.***   - Not applicable | Not applicable |
| 30. Statistical correction | ***Indicate whether any methods such as weighting of items or propensity scores have been used to adjust for the non‐representative sample; if so, please describe the methods.*** | Not applicable. |

This checklist has been modified from Eysenbach G. Improving the quality of Web surveys: the Checklist for Reporting Results of Internet E‐Surveys (CHERRIES). J Med Internet Res. 2004 Sep 29;6(3):e34 [erratum in J Med Internet Res. 2012; 14(1): e8.]. Article available at https://[www.jmir.org/2004/3/e34/;](http://www.jmir.org/2004/3/e34/%3B) erratum available http[s://w](http://www.jmir.org/2012/1/e8/)ww[.jmir.org/2012/1/e8/](http://www.jmir.org/2012/1/e8/). Copyright

©Gunther Eysenbach. Originally published in the Journal of Medical Internet Research, 29.9.2004 and 04.01.2012.

This is an open‐access article distributed under the terms of the Creative Commons Attribution License (https://creativecommons.org/licenses/by/2.0/), which permits unrestricted use, distribution, and reproduction in any medium, provided the original work, first published in the Journal of Medical Internet Research, is properly cited.

***References for Additional File 2***

1. Eysenbach, G., *Improving the quality of Web surveys: the Checklist for Reporting Results of Internet E‐Surveys (CHERRIES).* Journal of medical Internet research, 2004. **6**(3): p. e34‐e34.
2. Eysenbach, G., *Correction: Improving the Quality of Web Surveys: the Checklist for Reporting Results of Internet E‐Surveys (CHERRIES).* Journal of Medical Internet Research, 2012. **14**(1): p. e8.
3. Qualtrics, *Qualtrics Security White Paper ‐ defining procedures and operations for secure operations at Qualtrics*. 2014, Qualtrics: 400 W. Qualtrics Dr., Provo, Utah, United States.
4. Barnes, L.A.J., et al., *Using Facebook to recruit to a national online survey investigating complementary medicine product use in pregnancy and lactation: a case study of method.* Research in Social and Administrative Pharmacy, 2020.
