# Supplementary material for: Demographics, health literacy and health locus of control beliefs of Australian women who take complementary medicine products during pregnancy and breastfeeding: A cross‐sectional, online, national survey
Source: Health Expect. 2021 Dec 23;25(2):667–83. doi: 10.1111/hex.13414 (PMC8957740; doi:10.1111/hex.13414)
Supplement: Supplementary file 3 — Supporting information. [file HEX-25--s001.pdf]

Additional File 3. Additional presentation of results from the statistical analyses

Table AF3.1. Numbers of dietary supplements taken by the sample

**Number of dietary supplements taken**	**Number of respondents (n=810)**	**Relative frequency (%)**	**Number of herbal medicines taken**	**Number of respondents (n=810)**	**Relative frequency (%)**
1	133	18.5	1	127	35.9
2	161	22.5	2	93	26.3
3	156	21.8	3	67	18.9
4	96	13.4	4	36	10.2
5	74	10.3	5	8	2.3
6	37	5.2	6	12	3.4
7	16	2.2	7	7	2.0
8	13	1.8			
9	8	1.1			
10 or more	23	3.2			
Total	717	100			
Missing data	93				

Table AF3.2 Number of herbal medicines taken by the entire sample

**Number of herbal medicines taken**	**Number of respondents (n=810)**	**Valid %**
1	127	35.9
2	93	26.3
3	67	18.9
4	36	10.2
5	8	2.3
6	12	3.4
7	7	2.0
8 or more	11	3.1
Total	354	100.0
Missing data	456	

Table AF3.3. Results of the single item health literacy question (risk of inadequate health literacy) - How confident are you filling out medical forms by yourself? [1]

Response option	Pregnant respondents (n=354)	Relative frequency (%)	Breastfeeding respondents (n=456)	Relative frequency (%)	Total sample (n=810)	Relative frequency (%)
**Extremely**	248	74.7	315	78.9	563	77.0
**Quite a bit**	67	20.2	73	18.3	140	19.2
**Somewhat**	14	4.2	10	2.5	24	3.3
**A little bit**	3	0.9	0	0.0	3	0.4
**Not at all**	0	0	1	0.3	1	0.1
**Total**	332	100	399	100	731	100
**Missing data**	22		57		79	

Pearson Chi-Square χ^2 value = 6.814, p =0.146

Table AF3.4. Functional heath literacy [FHL] - Newest Vital Sign [2,3] results

	Pregnant respondents (n=354)	Relative frequency (%)	Breastfeeding respondents (n=456)	Relative frequency (%)	Total sample (n=810)	Relative frequency (%)
**Limited FHL**	14	4.2	10	2.5	24	3.3
**At risk of inadequate FHL**	9	2.7	16	4.0	25	3.4
**Adequate FHL**	308	93.1	374	93.5	682	93.3
**Total responses**	331	100	400	100	731	100
**Missing**	23		56		79	

Pearson Chi-Square χ^2 value = 2.523, p=0.283

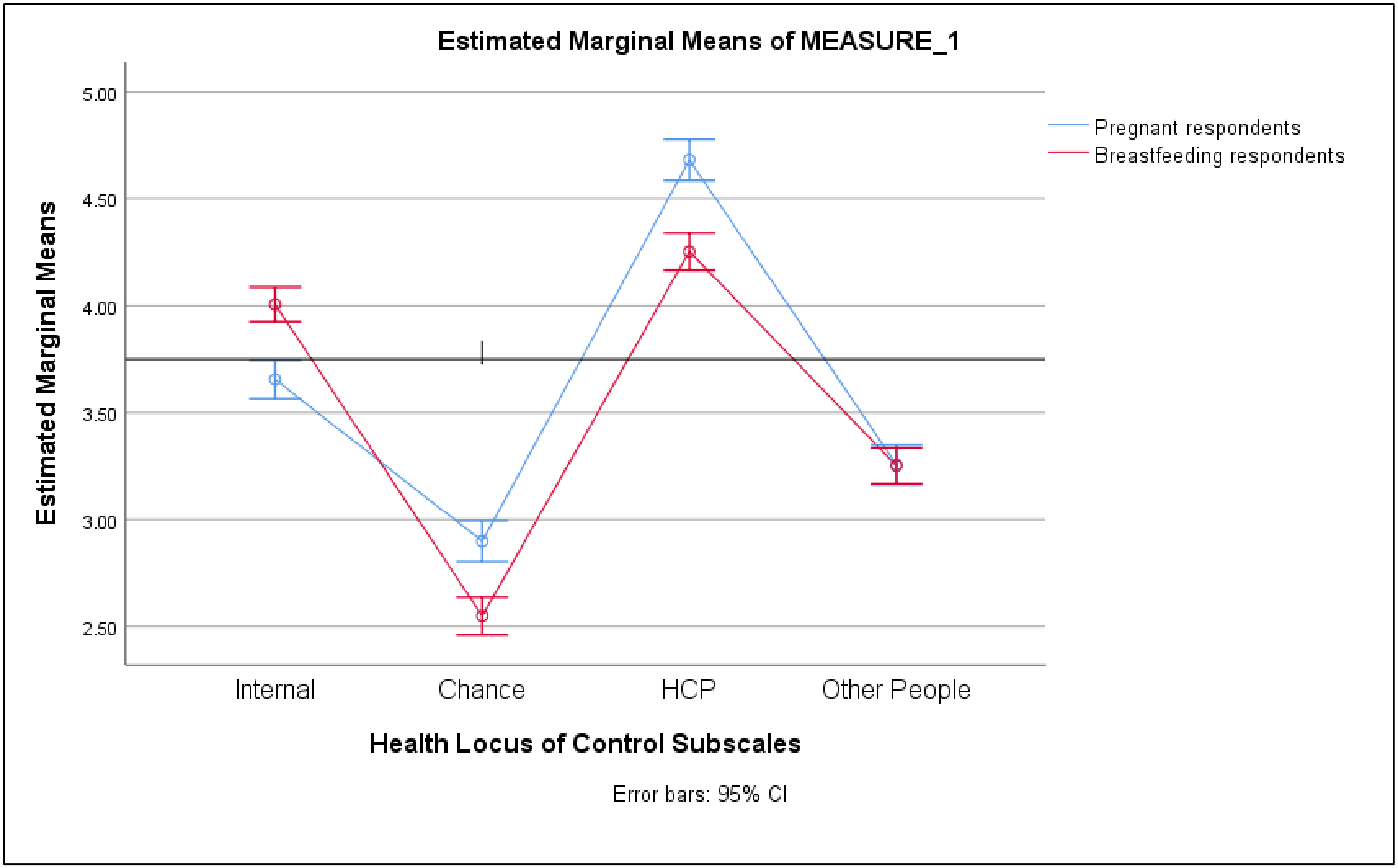

Figure AF3.1. Mean scores for the four sub-scales of the Health Locus of Control

Key: Internal = Internal Health Locus of Control [HLOC]; Chance = Chance HLOC; HCP = Health Care Practitioners HLOC; Other People = Other People HLOC.

References cited in Additional File 3

1. Wallace LS, Rogers ES, Roskos SE, Holiday DB, Weiss BD. Brief report: screening items to identify patients with limited health literacy skills. Journal of general internal medicine. 2006;21:874-7. doi: 0.1111/j.1525-1497.2006.00532.x.
2. Rowlands G, Khazaezadeh N, Oteng-Ntim E, Seed P, Barr S, Weiss BD. Development and validation of a measure of health literacy in the UK: the newest vital sign. BMC Public Health. 2013;13:116.
3. Weiss BD, Mays MZ, Martz W, Castro KM, DeWalt DA, Pignone MP, et al. Quick assessment of literacy in primary care: the newest vital sign. Annals of family medicine. 2005;3:514-22. doi: 10.1370/afm.405.
