# Supplementary material for: Demographics, health literacy and health locus of control beliefs of Australian women who take complementary medicine products during pregnancy and breastfeeding: A cross‐sectional, online, national survey
Source: Health Expect. 2021 Dec 23;25(2):667–83. doi: 10.1111/hex.13414 (PMC8957740; doi:10.1111/hex.13414)
Supplement: Supplementary file 4 — Supporting information. [file HEX-25--s004.pdf]

Additional File 4. Definitions of complementary medicine products from other High-Income Economies

The World Bank classifies Australia, the United Kingdom (UK), the United States of America (USA) and Germany as High Income Economies (HICs).[1] Comparisons of the operational definition of CMPs used in this research (Australian context), with the definitions of comparable products given by relevant government bodies from the UK, USA and Germany are shown below.

Country and term used	Definition includes		Other information
**Australia -** *Complementary medicine products (CMPs) as defined in the Australian context for this research.*	*Vitamins and Minerals*<br>Examples of supplements include vitamin or mineral supplements, fish oils, and probiotics.	*Herbal medicines*<br>Examples of herbal medicines include herbal teas, herbal extracts, tablets and capsules.	Some vitamins and minerals (e.g., iron, folate or iodine supplements) may be recommended by your doctor or other healthcare practitioner.<br><br>Other CMPs like some herbal medicines may have traditional uses but may not have been scientifically researched.
**USA**[2,3] – *Dietary supplements*	"Have one or more dietary ingredients, including vitamins, minerals, herbs or other botanicals, amino acids, enzymes, tissues from organs or glands, or extracts of these."	"Herbal supplements - sometimes called botanicals - are a type of dietary supplement containing one or more herbs."	"Federal law defines dietary supplements as products that:<br>• You take by mouth (such as a tablet, capsule, powder, or liquid)<br>• Are made to supplement the diet.<br>• Are labeled as being dietary supplements."
**UK**[4] – *Food supplements*<br>"any food the purpose of which is to supplement the normal diet and which is a concentrated source of a vitamin or mineral or other substance with a nutritional or physiological effect, alone or in combination and is sold in dose form".	"A wide range of nutrients and other ingredients might be present in food supplements. These can include<br>• vitamins<br>• minerals<br>• amino acids<br>• essential fatty acids<br>• fibre"	"A wide range of nutrients and other ingredients might be present in food supplements. These can include<br>• […]<br>• various plants and herbal extracts"	"Food supplements are intended to correct nutritional deficiencies, maintain an adequate intake of certain nutrients, or to support specific physiological functions. They are not medicinal products and as such cannot exert a pharmacological, immunological or metabolic action. Therefore, their use is not intended to treat or prevent diseases in humans or to modify physiological functions.<br>"In the UK, food supplements are required to be regulated as foods and are subject to the provisions of general food law."<br>"Regarding recommended dietary supplements in pregnancy and lactation like folic acid, vitamin D, and iron, the NHS (UK health dept) states: "You can get supplements from pharmacies and supermarkets, or a GP may be able to prescribe them for you."[5]

Additional File 4 for the paper:
Demographics, health literacy, and health locus of control beliefs of Australian women who take complementary medicine products during pregnancy and breastfeeding: a cross-sectional, online, national survey.

Germany[6] – *Food supplements* “Food supplements are foods. They are intended to supplement the general diet. Food supplements may contain a broad spectrum of nutrients or other substances with a specific nutritional or physiological effect.”	“They may be, for instance, vitamins, minerals, trace elements, amino acids, fatty acids, … which are added in concentrated form either individually or as a combination.”	“They may be, for instance, … fibre, plants or herbal extracts which are added in concentrated form either individually or as a combination.”	“Food supplements are sold in a dosed form, for instance as tablets, capsules, coated tablets, powders or liquids for intake in small, measured amounts.”<br><br>“For the purposes of improving or reliably ensuring the adequate intake of folic acid, women planning to have a baby, or pregnant women, are advised to add food supplements to their diet. The vitamin folic acid has a positive impact on the complete closing of the neural tube in the embryo and reduces the risk of neural tube defects like "split spine" (Spina bifida) in newborn babies.”[6]
World Health Organization[7] (herbal medicine definition)	--	“Herbal medicines include herbs, herbal materials, herbal preparations and finished herbal products, that contain as active ingredients parts of plants, or other plant materials, or combinations.”	---

References cited in Additional File 4

1. The World Bank. World Bank Country and Lending Groups. The World Bank Group. https://datahelpdesk.worldbank.org/knowledgebase/articles/906519-world-bank-country-and-lending-groups. Published 2021. Accessed Monday November 29th, 2021.
2. U.S. Food & Drug Administration. Information for Consumers on Using Dietary Supplements. U.S. Food and Drug Administration. https://www.fda.gov/food/dietary-supplements/information-consumers-using-dietary-supplements. Published 2019. Accessed November 26th, 2021, 2021.
3. National Center for Complementary and Integrative Health. Dietary and Herbal Supplements. U.S. Department of Health and Human Services, National Institute of Health. https://www.nccih.nih.gov/health/dietary-and-herbal-supplements. Published 2021. Updated November 29th, 2021. Accessed November 29th, 2021.
4. Food Standards Agency. Food supplements. Food Standards Agency [GOV.UK]. https://www.food.gov.uk/business-guidance/food-supplements. Published 2021. Updated 24 March 2021. Accessed November 24th, 2021.
5. National Health Service (NHS). Vitamins, supplements and nutrition in pregnancy. NHS. https://www.nhs.uk/pregnancy/keeping-well/vitamins-supplements-and-nutrition/. Published 2021. Updated 14 February 2020. Accessed Accessed November 26th, 2021.
6. Bundesinstitut für Risikobewertung (BfR). Frequently Asked Questions on Food Supplements. Bundesinstitut für Risikobewertung (BfR). https://www.bfr.bund.de/en/frequently_asked_questions_on_food_supplements-70347.html. Published 2021. Updated 4 December 2018. Accessed Accessed November 26th, 2021.
7. World Health Organisation. Traditional, complementary and integrative medicine. http://www.who.int/traditional-complementary-integrative-medicine/about/en/. Published 2021. Accessed November 25th, 2021.
